# Seasonal malaria chemoprevention packaged with malnutrition prevention in northern Nigeria: A pragmatic trial (SMAMP study) with nested case-control

**DOI:** 10.1371/journal.pone.0210692

**Published:** 2019-01-25

**Authors:** Abigail Ward, Andrea Guillot, Lyudmila E. Nepomnyashchiy, Justin C. Graves, Kathleen Maloney, Omowunmi F. Omoniwa, Leslie Emegbuonye, Charles Opondo, Marko Kerac, Elizabeth Omoluabi, Antoinette Bhattacharya, Karen Milch Hariharan, Owens Wiwa, Justin M. Cohen, Arnaud Le Menach

**Affiliations:** 1 Clinton Health Access Initiative, Boston, Massachussetts, United States of America; 2 Department of Medical Statistics, London School of Hygiene & Tropical Medicine, London, England; 3 Department of Population Health, London School of Hygiene & Tropical Medicine, London, England; 4 Akena, Abuja, Nigeria; Centers for Disease Control and Prevention, UNITED STATES

## Abstract

Integrating seasonal malaria chemoprevention (SMC), recommended by the WHO since 2012 to prevent malaria infection, with nutrition interventions may improve health outcomes and operational efficiencies. This study assessed the effects of co-packaging interventions on distribution coverage, nutrition, and clinical malaria outcomes in northern Nigeria. From August to November 2014, community volunteers delivered sulfadoxine-pyrimethamine and amodiaquine (SP-AQ) door-to-door each month to approximately 7,000 children aged 6–24 months in seven wards of Madobi, Kano State, Nigeria. In three of the wards children additionally received a lipid-based nutrient supplement (LNS–medium quantity), Plumpy Doz. Coverage, adherence, and anthropometric outcomes were assessed through baseline, midline, and endline household surveys. A facility-based case-control study was also conducted to estimate impact on clinical malaria outcomes. Coverage of SP-AQ was similar between arms at 89% (n = 2,409 child-months [88–90%]) in the SP-AQ only arm and 90% (n = 1,947 child-months [88–92%]) in the SP-AQ plus LNS arm (p = 0.52). Coverage of LNS was 83% (n = 2,409 child-months [81–84%]). Whilst there were marked changes in anthropometric status between baseline, midline and endline, these were largely accounted for by socioeconomic status and must be interpreted with care due to possible measurement issues, especially length-based indices. Overall nutritional status of our most robust measure, weight-for-age, does appear to have improved by endline, but was similar in the two study arms, suggesting no additional benefit of the LNS. While the odds of clinical malaria among those who received the intended intervention were lower in each study arm compared to children who did not receive interventions (SP-AQ only OR = 0.23 [0.09–0.6]; SP-AQ plus LNS OR = 0.22 [0.09–0.55]), LNS was not shown to have an additional impact. Coverage of SMC was high regardless of integrating LNS delivery into the SMC campaign. Supplementation with LNS did not appear to impact nutritional outcomes, but appeared to enhance the impact of SP-AQ on clinical odds of malaria. These results indicate that combining nutritional interventions with seasonal malaria chemoprevention in high-risk areas can be done successfully, warranting further exploration with other products or dosing.

Trial Registration: ISRCTN 11413895

## Introduction

Malnutrition and malaria are priority challenges for global child health. Nutritional disorders are implicated in over three million deaths in children under five annually (45% of total child mortality) while malaria contributed to the deaths of 306,000 children in 2015 [1,2]. In Africa, 36% of children under five years old are stunted and 18% are underweight, while 16% of children between two and ten years of age are infected with malaria parasites [1,2].

African children at risk for malaria are often also at risk for malnutrition. While the relationship between malnutrition and infectious disease is accepted to be generally synergistic, the specific malnutrition-malaria interaction is not well-defined [3–5]. Risk of malaria in stunted children may be increased by immune system inhibition [6,7], and several studies indicate that malaria infection in underweight and wasted children amplifies case fatality by up to ninefold [8–11]. At the same time, anemia and immune suppression resulting from malaria infection contributes to or may be exacerbated by malnutrition [9,12–15]. Other studies show conflicting results, with no association between infection and nutrition status or increased incidence of malaria only in either underweight or stunted children [4,10,16–19].

Interventions targeting both acute malnutrition and infectious disease may therefore act synergistically to improve overall child health. Previous research has shown that integrating community interventions for multiple diseases increases coverage, improves health outcomes, and is cost-effective [20–22]. In northern Nigeria, malnutrition and malaria are both highly seasonal, spiking during the rainy season, lending them to an integrated approach. Malnutrition rates are particularly high in the North West Zone, where Kano State is located: as of Nigeria’s 2013 Demographic and Health Survey, 27% of under-five children in the North West Zone and 40% of under-five children in Kano State suffered from severe acute malnutrition or wasting [23]. In Kano, malaria prevalence among children under five was 60% in the 2015 Malaria Indicator Survey [24]. Combining resource-intensive community-directed intervention campaigns for malnutrition and malaria may therefore provide a cost- and operationally effective opportunity.

Seasonal malaria chemoprevention (SMC), in which children under five years are given a monthly dose of anti-malarial drugs, is recommended since 2012 by the World Health Organization (WHO) in certain areas where malaria transmission is highly seasonal, such as the Sahel sub-region of Africa [25]. SMC has been shown to reduce uncomplicated and severe malaria episodes by 75% [26]. It is typically delivered door-to-door or through fixed or mobile health service points and administered by community health workers. An estimated 6.4 million children received SMC in 2016 [27].

SMC campaigns may provide an opportunity for delivery of an integrated package of child health interventions. Medium-quantity lipid-based nutrient supplements (LNS-medium quantity), such as Plumpy Doz, are used in food-insecure settings to provide additional calories, protein, fats, and micronutrients to young children who are moderately malnourished and/or at risk for severe acute malnutrition. While studies of the effect of LNS on anthropometric indicators have found mixed results, provision of LNS to children at risk of undernutrition has improved weight gain, cognitive function, and hemoglobin concentration in some settings [28–32]. Conveniently packaged as a ready-to-use food, LNS has been widely accepted by caretakers and children [33–35]. Integrating LNS with SMC campaigns may thus offer an opportunity to achieve additional improvements in child health beyond those achieved by distribution of malaria chemoprevention alone. Therefore, in this study the aim was to determine whether intervention coverage, child nutrition, and health outcomes are improved by packaging the delivery of LNS through the SMC campaign compared with the delivery of SMC alone.

## Methods

This study was designed as a pragmatic cluster controlled trial with a nested case-control. Delivery of SMC combined with LNS was evaluated against delivery of SMC alone from August 2014 to May 2015 in Madobi Local Government Area (LGA) of Kano State, Nigeria. Madobi had an estimated population of 11,000 children under five years old in 2014, using projected figures from the 2006 National Census [36]. The study was conducted in two non-adjacent intervention arms consisting of seven wards (Yakun, Rikadawa, Kubaraci, Kafin Agur, Burji, Kanwa, and Kauran Mata) (S1 Fig). The wards included in the study had similar characteristics, including malaria endemicity (*P*. *falciparum* prevalence among 2–10 year olds in 2014 was 30.4% in the SP-AQ only area and 28.4% in the SP-AQ plus LNS area) and seasonality (the two intervention areas are separated by approximately 4 km) [37]. Density of health facilities was similar (6 in each study arm), and a Community-based Management of Acute Malnutrition (CMAM) facility was present in each study area. Both study areas are rural, and most families in the study area are involved in agriculture, growing a variety of crops that includes maize, rice, millet, groundnuts, potatoes, soybeans, and beans. Irrigation is not standard but is present.

### Selection and description of participants

Children aged 6 to 24 months as of August 2014 living in the study wards were eligible to participate in the trial. Children were excluded if: they had an allergy to any component of the intervention; had taken SP or AQ within 30 days of intervention delivery; were HIV-positive and taking co-trimoxazole; were unable to take oral medication; had suffered a severe acute illness including severe acute malnutrition (SAM), or had confirmed or suspected malaria at the time of the household visit. Any child with severe acute illness or high fever was referred to the nearest public health facility.

Children in the study population who sought care for fever in one of the 13 primary health care facilities in the seven intervention wards between August 28 and November 30, 2014 were also eligible for recruitment in the unmatched health facility-based case-control study. Children not eligible for the intervention (SP-AQ or SP-AQ plus LNS) were excluded as well as children referred to a CMAM facility in the past month or diagnosed with severe acute malnutrition (mid-upper arm circumference (MUAC) <11.5cm). A case was defined as any child from the study population with a current or reported history of fever of ≥37.5 C within the past 48 hours with a positive malaria rapid diagnostic test (mRDT). A control was defined as a child from the study population with a current or reported history of fever of ≥37.5 C within the past 48 hours and a negative mRDT test.

### Interventions

The seven study wards were split into two groups based on geographic proximity. Children in the northern wards of Kafin Agur, Burji, Kanwa, and Kauran Mata received SP-AQ only, while children in the southern wards of Kubaraci, Rikadawa, and Yakun received SP-AQ and LNS. The group of wards, or arm, receiving the LNS intervention was randomly selected. Following community sensitization activities, a monthly SMC campaign of WHO pre-qualified, co-blistered sulfadoxine-pyrimethamine plus amodiaquine (SP-AQ, brand name SPAQ-CO, Guilin Pharmaceuticals) was delivered door-to-door in all seven wards by the Kano State Ministry of Health (KSMOH) network of Community-Directed Distributors (CDDs) for four consecutive months from August to November 2014. Timing of delivery coincided with the typical malaria season and the period before November harvest when food is most scarce. Children aged 6 months to 11 months received SP 262.5mg + AQ 75mg, while children aged 12 to 24 months received SP 525mg + AQ 150mg. All drugs were formulated as a three-day course of tablets. All CDDs were trained in directly observed therapy (DOT) administration and remained at households for 15 minutes following ingestion of the first dose of SP-AQ (and LNS if applicable) to monitor for adverse reactions. Parents/guardians were given the remainder of the treatment courses with administration instructions.

In one of the two groups of wards, the southern group of Kubaraci, Rikadawa, and Yakun,each monthly SP-AQ distribution was bundled with a one-month supply of the LNS product Plumpy’Doz (Nutriset). Neither the deliverers of the intervention nor the outcome assessors were blinded to the group allocations. For LNS, caregivers were instructed to feed children aged 6–11 months the recommended dose of 23 grams per day, or 1.5 teaspoons three times per day, while children 12–24 months received 46 grams per day, or three teaspoons three times per day (in addition to breastmilk and other available foods) [38]. A daily dose of 46 grams of Plumpy’Doz contains 9.0mg of iron and is in accordance with WHO recommendations on iron supplementation in malaria-endemic areas [39,40].

### Ethics statement

The Kano State Hospitals Management Board (KSHMB Reference #HMB/GEN/488/VOL. I) approved all study protocols. Data collectors obtained written informed consent from a parent or guardian aged 18 years or older for all study participants via signature, or fingerprint for non-literate participants. The Kano State Hospitals Management Board approved the consent procedure.

### Evaluation

**Fig 1:**
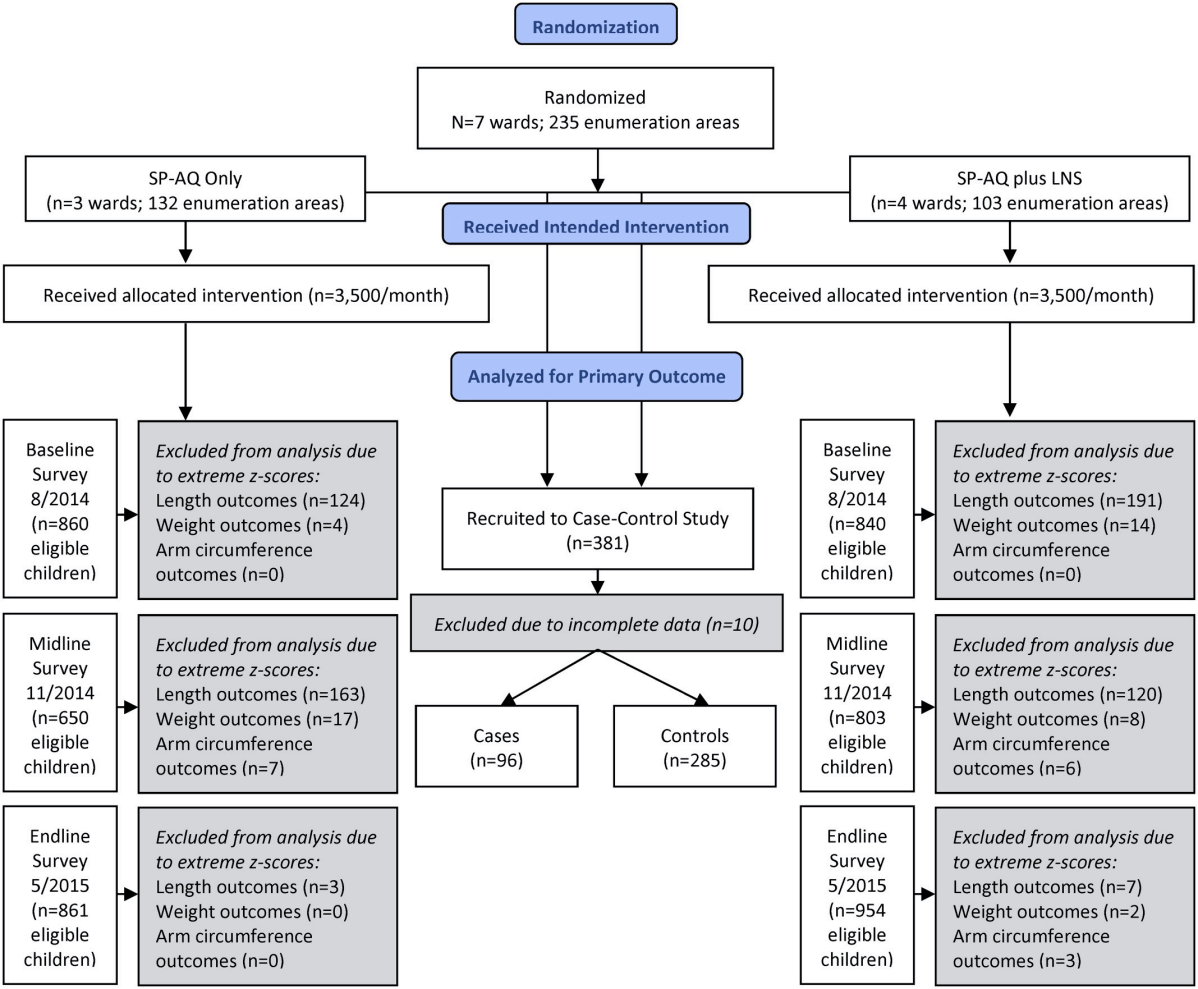
Participant flow diagram of trial participants with three cross-sectional surveys and a nested case-control study.

Coverage, adherence, and nutrition outcomes were measured using repeated cross-sectional household surveys. Three household surveys were conducted: prior to the first round of distribution in August 2014 among children aged 6–24 months (baseline), following the last round of distribution in November 2014 among children aged 9–27 months (midline), and six months after the last round of distribution among children 15–33 months old (endline) (Fig 1). An electronic questionnaire was administered face-to-face in Hausa with the head of household or caregivers using Open Data Kit version 1.4.4 (baseline and midline) and SurveyCTO v1.31 (endline). Information was collected on demographics and household characteristics, household food insecurity, recent illness/fever history, breastfeeding practices, and receipt of and adherence to SP-AQ and LNS interventions. Anthropometric measurements (length, weight, and MUAC) were also collected from children using UNICEF height boards and infantometers (accurate to 0.1 cm), Seca scales (accurate to 0.05 kg) and MUAC tapes (accurate to 0.1 cm). Enumerators performed each measurement twice, and if the difference was greater than 10%, a third measurement was taken. The final measure was defined as the mean of the two measurements, or the mean of the two closest measurements with the most extreme measure excluded if a third measure was taken.

For the case-control study, each of the 13 primary health care facilities in the study area participated in a training based on national guidelines, covering mRDT performance and safety, recruiting cases and controls, and completing study questionnaires. Facilities were supplied with mRDTs during the study. Information on the cases and controls was collected from caregivers by a healthcare worker using a register book, including: age and gender, history of disease including current and previous symptoms, mRDT results, recall of interventions received in the last 30 days (SP-AQ only or SP-AQ plus LNS), and other malaria prevention measures such as insecticide-treated net (ITN) use or indoor residual spraying (IRS) within the home.

### Sample size calculations

Selection of households for surveys was done using a two-step design with enumeration areas delineated for the 2006 National Census as the primary sampling unit, and households as the secondary sampling unit. Using ward-level population estimates, a probability-proportional-to-size calculation determined how many enumeration areas to include from each ward to obtain a sample of 750 households from each intervention area (S1 Table). Simple random sampling without replacement was done to select thirty enumeration areas per arm from a total of 132 enumeration areas across the four wards in the SP-AQ only area and 103 enumeration areas across the three wards in the SP-AQ plus LNS area. Within each selected enumeration area, 25 households were selected using a listing of all eligible households within the enumeration area. Household eligibility was defined as at least one child aged 6–24 months in August 2014 within the household. All children aged 6 to 24 months in August 2014 in selected eligible households were included in the survey. This sample is sufficient to demonstrate a 10% absolute reduction in each outcome with 90% power at the 5% level of significance, assuming 50% prevalence at baseline and design effect of 1.5.

Sampling weights were calculated based on the MEASURE DHS approach for design and sampling weights [41]. Weights were calculated as the inverse of the combined probability of an enumeration area being selected from a ward and the probability of a household with at least one child aged 6–24 months in August 2014 being selected from an enumeration area.

The case-control study aimed to include 604 controls and 302 cases, based on a ratio of 2 controls:1 case, assuming 60% intervention coverage, a detectable odds ratio of 0.6 of receiving SP-AQ plus LNS compared with SP-AQ only, and an average of 6 confirmed malaria cases per facility per month (two-tailed p<0.05, power = 0.8).

### Data analysis

Primary outcomes were intervention coverage and adherence, nutritional status of children, and clinical malaria incidence (Table 1). Analysis was conducted according to randomized group allocation, regardless of whether the assigned intervention was received (i.e. intention-to-treat). While all eligible children 3–59 months old in August 2014 received SMC as per programmatic recommendations, only children 6–24 months of age in August 2014 were included in nutrition interventions and evaluation [40]; therefore, malaria outcomes were also restricted to study participants ages 6–24 months at baseline. Because SP-AQ is considered effective for four weeks after administration, each month of protection for each child is considered for coverage calculations (four months per child per malaria season) [25]. Coverage was defined as the proportion of child-months over which eligible children were protected out of all eligible child-months, measured during the midline survey through a series of survey questions about receipt and consumption of SP-AQ during each month of delivery. Adherence to SP-AQ was measured at the midline survey as the proportion of children who took the full three-day treatment course in the final round of distribution. Adherence to LNS was measured as the proportion of children who received LNS in the final round of distribution and consumed it in the 24 hours prior to the survey. Nutritional status was measured by the prevalence of stunting, wasting, and underweight, and change in z-scores for length-for-age (LAZ), weight-for-length (WLZ), weight-for-age (WAZ) and MUAC across survey rounds. Nutrition outcomes were also compared between intervention arms at each time point.

**Table 1:** Summary of study outcomes, variable sources, and comparison populations.

Outcome	Evaluation	Comparison
**Objective 1: Compare coverage and adherence to interventions**		
Percentage of children receiving intervention	Household survey (midline)	Intervention groups at end of distribution
Intervention adherence	Household survey (midline)	Intervention at end of distribution
**Objective 2: Evaluate change in nutrition outcomes**		
Change in prevalence of stunting (LAZ), underweight (WAZ), and wasting (WLZ and MUAC)	Household surveys	Intervention groups and time
Change in mean z-scores (LAZ, WAZ, WLZ, MUAC)	Household surveys	Intervention groups and time
**Objective 3: Evaluate change in malaria outcomes**		
Odds of clinical malaria	Case-control	Intervention groups

Z-scores were generated using the WHO Anthro macro for Stata, which allows for comparison of anthropometric measures with 2006 WHO Child Growth Standards [42]. Stunting was defined as LAZ less than -2, wasting as WLZ less than -2 and underweight as WAZ scores of less than -2. Low MUAC was defined as MUAC of less than 11.5 centimeters and severe acute malnutrition (SAM) as WLZ scores of less than -3 and/or low MUAC. Extreme individual LAZ (less than -6 or greater than 6), WAZ (less than -6 or greater than 5), WLZ (less than -5 or greater than 5), and MUAC z-scores (less than -5 or greater than 5) were excluded; valid z-scores for the same child were kept [42]. Although the standard definition of SAM also requires assessment for oedema, this sign was not assessed and is therefore not part of the SAM definition in this project. Household food insecurity covariates were created using the standardized methodology from the Food and Nutrition Technical Assistance Project (FANTA) question series [43]. A wealth index was constructed from household asset data using principal components analysis [44,45]. Food insecurity was compared between intervention arms and time points. Effects were expressed as weighted differences in z-scores and odds ratios for stunting, wasting, and underweight.

Evidence for a difference in coverage and adherence between arms was assessed using a Wald test. The test statistic was obtained by dividing the difference in coverage or adherence by the cluster-adjusted standard error of the difference and comparing the resulting test statistic to a standard normal distribution. The means and proportions were weighted to reflect the sampling design and standard errors were adjusted for clustering within enumeration areas using the Huber-White “sandwich” estimator of robust standard errors [46,47]. Differences in nutrition outcomes and clinical malaria incidence were assessed using hierarchical multivariable regression models adjusting for clustering within enumeration area. Mean z-score and malnutrition prevalence regression models included intervention arm, survey (time), and interaction parameters for intervention arm and time as the measure of the potential additional effect of the intervention at midline and endline relative to baseline. All models were adjusted for demographics, household food insecurity, and wealth.

A logistic regression model was used to compare the impact of intervention (SP-AQ or SP-AQ plus LNS versus no intervention), study arm, and the interaction between intervention and study arm on the odds of clinical confirmed malaria in cases versus controls, taking into account clustering at the health facility. The model adjusted for demographics, month of recruitment, illness in the two weeks prior to enrollment, and ITN use (S4 Table). Analysis was restricted to children who received the intended intervention (SP-AQ in the SP-AQ only arm; SP-AQ plus LNS in the SP-AQ plus LNS arm). All analyses were performed using Stata 13 (StataCorp) with significance defined as p<0.05.

## Results

Approximately 7,000 children aged 6–24 months as of August 2014 (3,500 in each intervention arm) were eligible to receive an intervention (Fig 1). Over the four months of intervention, 40 adverse reaction episodes to SP-AQ were reported across the seven study wards (38 vomiting/spitting up, 2 fever).

### Cross-sectional survey results

There were 1,492 households at baseline, 1,220 households at midline, and 1,579 households at endline with children 6–24 months old as of August 2014 (Fig 1). The midline survey inadvertently included households with children 3–63 months old; 77% of the 1,583 surveyed households met the eligibility requirement of at least one child 6–24 months old in August 2014 and were included in the analysis.

A total of 4,968 children were sampled from the 4,291 households across both intervention groups over the three surveys (Fig 1). Households in the two intervention arms were well balanced with respect to household composition and size, as well as demographic characteristics (education, religion, and ethnicity) and self-reported food security (Table 2, S2 Table). There was a difference in household wealth observed in all three surveys with the SP-AQ only area having significantly more households in higher wealth quintiles in all three survey rounds (Chi-square p = 0.05 at baseline, p = 0.02 at midline, p = 0.06 at endline).

**Table 2:** Household survey sample sizes and demographic characteristics of respondents at baseline, midline, and endline.

	Baseline		Midline		Endline	
	*SP-AQ Only*	*SP-AQ+LNS*	*SP-AQ Only*	*SP-AQ+LNS*	*SP-AQ Only*	*SP-AQ+LNS*
**Child age**	6–24 months		9–27 months		15–33 months	
**Number of children**	860	840	650	803	861	954
**Number of households**	751	741	656	564	775	804
**Respondent is Head of Household (proportion of households, 95% CI)**	68.0% (56.9–79.1)	73.4% (65.6–81.4)	69.5% (62.3–76.7)	69.3% (61.9–76.7)	70.8% (64.4–77.2)	69.7% (62.2–77.2)
**Respondent is Primary Caregiver (proportion of households, 95% CI)**	38.5% (26.8–50.1)	35% (25.8–44.2)	43% (32.1–53.8)	44.8% (37.1–52.4)	39.7% (29.6–49.8)	39.8% (32.1–47.5)
**Wealth Index Quintiles (proportion of households, 95% CI, adjusted to baseline)**						
**Lowest**	16.8% (8.5–25.2)	22% (13.4–30.6)	10.9% (6.2–15.7)	20.7% (12.9–28.5)	14.5% (8.8–20.2)	22.9% (15.8–29.8)
**Lower**	16.3% (10.8–21.8)	24.4% (20.0–28.9)	14.8% (10.7–18.8)	18.7% (13.9–23.5)	16.4% (10.1–22.6)	16.3% (12.2–20.4)
**Middle**	17.6% (13.6–21.6)	22.1% (17.5–26.6)	26.3% (21.8–30.7)	25.1% (20.5–30.0)	20.1% (15.3–24.9)	24.2% (19.4–29.0)
**Higher**	23.3% (18.0–28.5)	16.5% (12.3–20.7)	20.9% (17.4–24.4)	17.6% (12.0–23.1)	23.1% (17.8–28.4)	20.1% (14.1–26.0)
**Highest**	26% (17.1–34.9)	14.9% (8.6–21.3)	27.7% (21.0–34.3)	18.3% (12.3–24.2)	25.9% (16.6–35.3)	17% (11.1–22.9)
**Household Self-Reported Food Secure**	65.7% (54.0–75.7)	66.5% (59.5–72.8)	64.6% (55.9–72.5)	68.2% (59.1–76.1)	64.1% (54.4–72.8)	61.2% (52.0–69.5)

**Fig 2:**
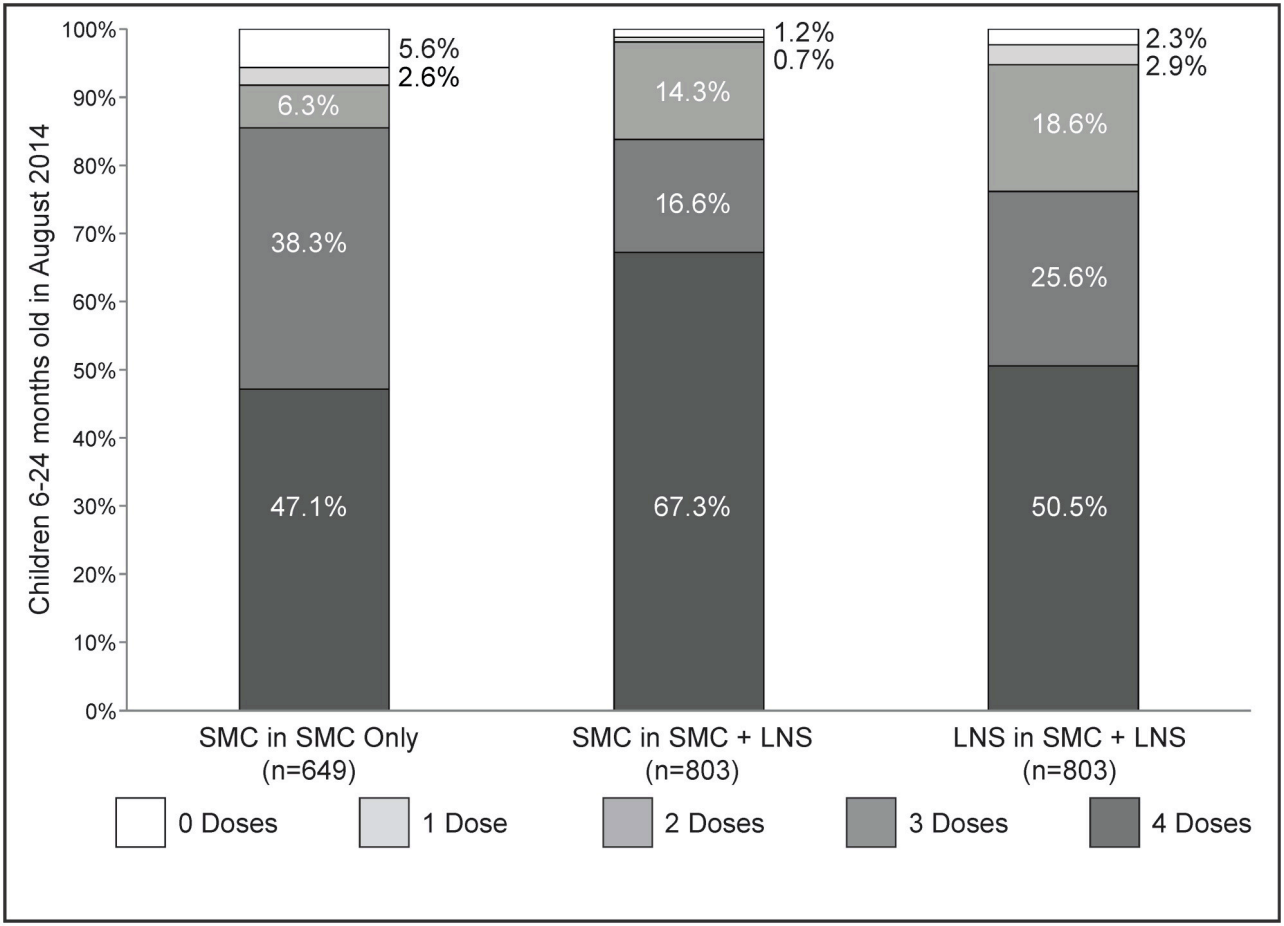
Distribution of the reported number of doses of SP-AQ and LNS received by intervention arm between August-November 2014, among all surveyed children aged 6–24 months as of August 2014. Each column totals 100%. The SP-AQ only area encompassed 132 enumeration areas while the SP-AQ plus LNS area included 103 enumeration areas.

Coverage and adherence results (Table 1, Objective 1): Among children 6–24 months old in August 2014, SP-AQ was received in 89.2% (n = 2,409 child-months; 95% CI 87.6–90.1%) of all possible child-months across the four delivery rounds in the SP-AQ only area and 89.8% (n = 1,947 child-months; 95% CI 87.7–91.8%) in the SP-AQ plus LNS area. There was no evidence of a difference in coverage between arms (p = 0.52). Coverage of LNS, measured by the proportion of potential child-months LNS received, was 82.5% (n = 2,409 child-months; 95% CI 80.9–84.8%) in the SP-AQ plus LNS area, and both SP-AQ and LNS were received in 80.7% (n = 2,409 child-months; 95% CI 78.6–82.8%) of child-months in this area. Coverage by number of treatments received is shown in Fig 2 and S3 Table.

Adherence to SP-AQ was measured at the midline survey as the proportion of children who completed the 3-day treatment course among children who received SP-AQ in the last distribution round. Reported adherence to the 3-day SP-AQ course was 83.8% (n = 369; 95% CI 74–90.4%) in the SP-AQ only area and 77.5% (n = 708; 95% CI 68.6–84.4%) in the SP-AQ plus LNS area (Wald test for difference in proportions between arms p-value = 0.27).

Adherence to LNS was also measured immediately following the final distribution round. Among children 6–24 months old as of August 2014 who were visited by a CDD in the fourth distribution round, 56.9% (n = 749; 95% CI 45.3–68.5%) reportedly consumed it in the 24 hours prior to the midline survey. Respondents of children who consumed LNS were also asked how much was fed per dose. Among children 6–9 months of age during the intervention, the median dose reported was 6 teaspoons (n = 53; range 1–20 teaspoons); among children 10–24 months the median daily consumption was 7 teaspoons (n = 335; range 1–19 teaspoons). During the endline survey, respondents were asked to recall whether any doses of LNS had been missed; reported complete treatment (no doses missed) was reported for 80.6% (n = 111; 95% CI 69.3–91.1%) of children 6–9 months old during the intervention and 83.7% (n = 532; 95% CI 75–92.5%) of children 10–24 months old during the intervention.

Nutrition outcome results (Table 1, Objective 2): Nutrition indicators were measured during all three surveys. In accordance with WHO protocols for anthropometric data analysis, 18.5% of children at baseline, 19.4% of children at midline, and 0.5% of children at endline were excluded from stunting outcomes due to extreme length-for-age z-scores. All of these were due to biologically implausible low LAZ: range at baseline (-6.01 to -21.82), midline (-6.02 to -16.17), and endline (-6.03 to -14.19). Underweight outcomes excluded 1.1% of the baseline sample, 1.7% of the midline sample, and 0.1% of the endline sample with extreme weight-for-age z-scores. Extreme weight-for-length z-scores were excluded from wasting outcomes (4.4% at baseline, 14% at midline, and 0.1% at endline), and extreme arm circumference z-scores were excluded from MUAC outcomes (0% at baseline, 0.9% at midline, 0.2% at endline (Fig 1).

Baseline stunting prevalence (LAZ<-2) was 78.9% (n = 736; 95% CI 73–83.8) in the SP-AQ only arm and 74.9% (n = 649, 95% CI 68.9–80.1%) in the SP-AQ plus LNS arm. Underweight prevalence (WAZ<-2) was 55.1% (n = 856; 95% CI 49.8–60.2%) in the SP-AQ only arm and 51.5% (n = 826; 95% CI 43.5–59.5%) in the SP-AQ plus LNS arm at baseline, and wasting prevalence (WHZ<-2) was 15.1% (n = 785; 95% CI 11.9–18.9%) in the SP-AQ only arm and 13.8% (n = 745; 95% CI 10.1–18.4) in the SP-AQ plus LNS arm at baseline. Low MUAC baseline prevalence (<11.5 cm) was 3.8% (n = 860; 95% CI 2–7%) in the SP-AQ only arm and 5.7% (n = 840; 95% CI 3.9–8.4%) in the SP-AQ plus LNS arm. At baseline, more than 1 in 10 children in the study area had severe acute malnutrition as indicated by WHZ<-3 and/or low MUAC: 10.1% (n = 860; 95% CI 7–14.4%) of children in the SP-AQ only arm and 11.6% (n = 840; 95% CI 8.5–15.6%) of children in the SP-AQ plus LNS arm.

**Fig 3:**
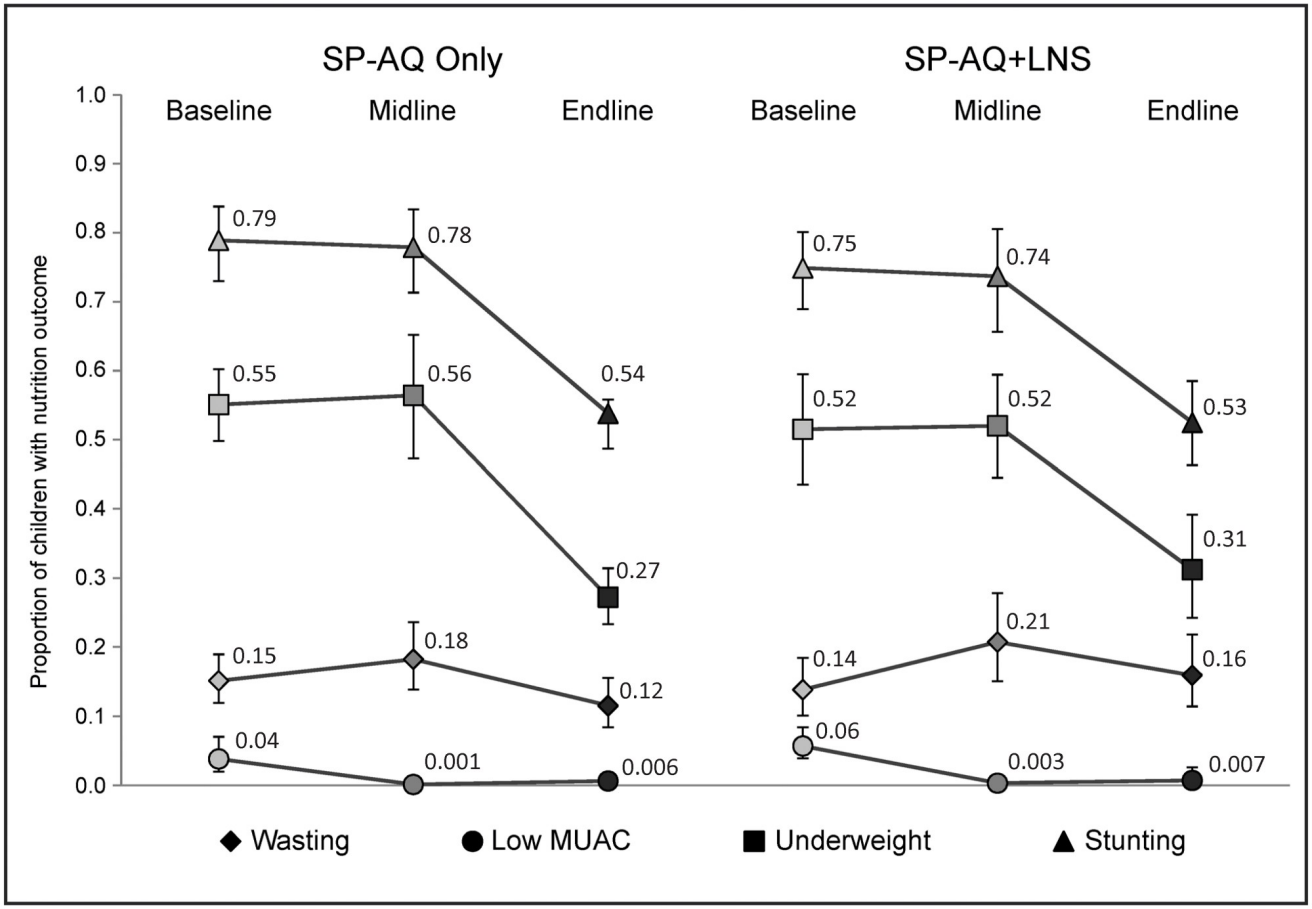
Prevalence of malnutrition indicators by intervention arm and survey for children 6–24 months as of August 2014 who received four doses of SP-AQ in the SP-AQ only arm or four doses of SP-AQ and four doses of LNS in the SP-AQ plus LNS arm.

**Fig 4:**
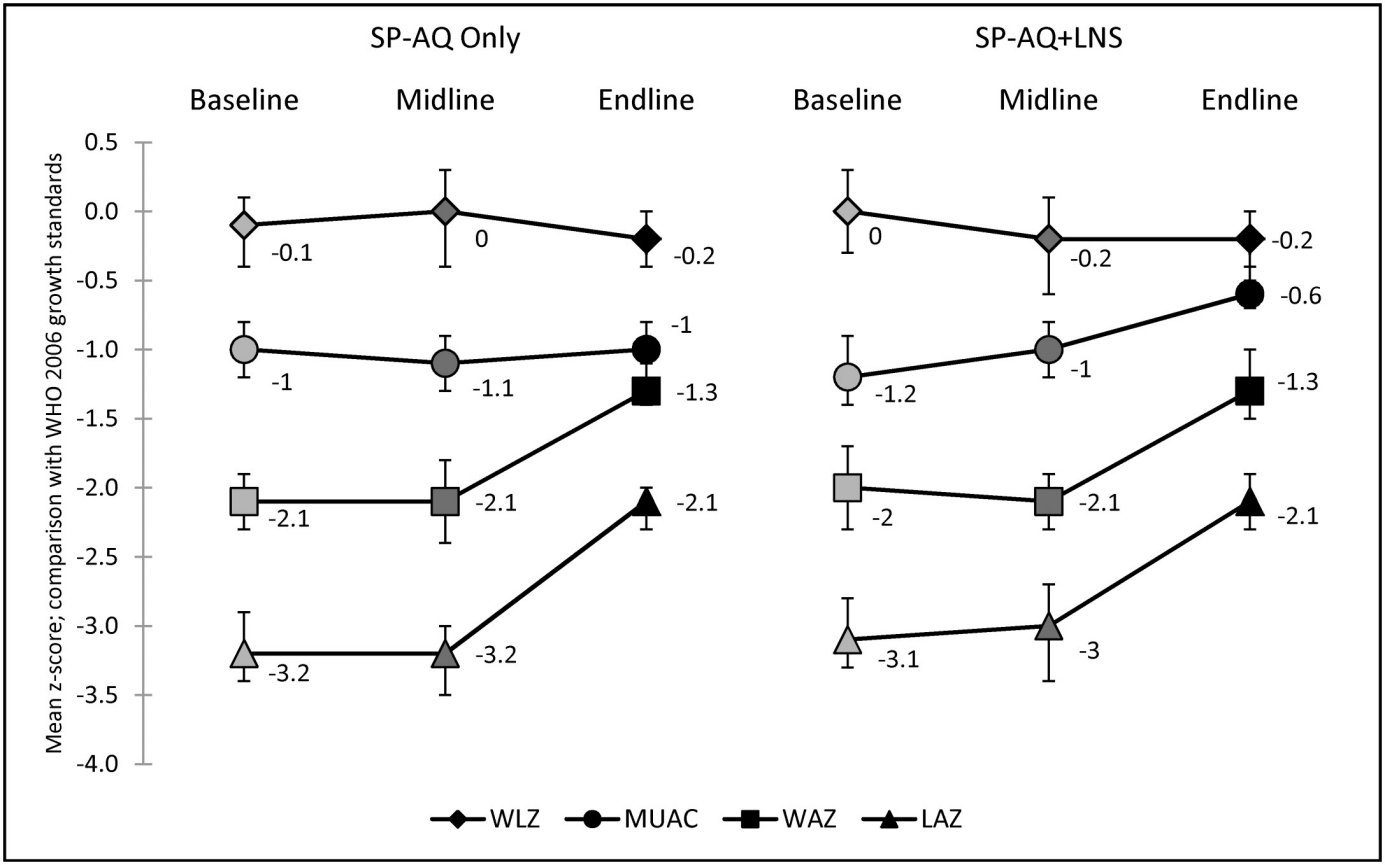
Mean z-scores for anthropometric outcomes. Denominators vary by indicator due to outlier exclusions (see S4 Table). Midline and endline results are limited to children who received four rounds of interventions.

Between baseline and endline, prevalence of stunting, underweight, low MUAC, and severe acute malnutrition were significantly lower within both intervention arms (p<0.01 for tests of difference over time in all areas and indicators), while prevalence of wasting was similar in both study arms at baseline and endline (S4 Table). Comparing intervention arms, there was no evidence of differences in prevalence of nutrition outcomes at baseline (Table 3). There was no evidence of differences in nutrition outcomes at midline or endline either, comparing children who received four rounds of SP-AQ only with children who received four rounds of SP-AQ in addition to four rounds of LNS (Fig 3). In accordance with changes in prevalence, mean LAZ and WAZ z-scores were higher at endline compared to midline in both intervention arms (Fig 4 and S4 Table).

**Table 3:** Multivariable regression outcomes for impact of the nutrition intervention. Coefficients represent the difference in anthropometric indicators comparing children 6–24 months as of August 2014 in each intervention arm across surveys (difference between arms in difference across time). Models are adjusted for household wealth.

	Midline-Baseline Comparison		Endline-Baseline Comparison		
**Mean Z-Score (Continuous Outcome)**	**Adjusted Difference in z-score**	**95% CI**	**Adjusted Difference in z-score**	**95% CI**	**p-value**
Length-for-age z-score	0.16	-0.44–0.76	0.01	-0.39–0.42	0.83
Weight-for-length z-score	-0.41	-1.01–0.20	-0.25	-0.71–0.21	0.38
Weight-for-age z-score	-0.08	-0.57–0.42	-0.14	-0.53–0.26	0.78
MUAC z-score	0.26	-0.12–0.64	0.60	0.26–0.94	<0.01
**Prevalence (Binary Outcome)**	**Adjusted Odds Ratio**	**95% CI**	**Adjusted Odds Ratio**	**95% CI**	**p-value**
Stunting (LAZ <-2)	0.94	0.45–1.97	1.18	0.70–1.98	0.67
Wasting (WLZ <-2)	1.35	0.69–2.62	1.69	0.90–3.18	0.26
Underweight (WAZ <-2)	0.97	0.52–1.80	1.43	0.87–2.33	0.25
Low MUAC (<115mm)	1.93	0.17–22.41	0.82	0.15–4.59	0.84

Household wealth was the only variable included in the final model due to an imbalance between arms at baseline. After adjustment for household wealth in multivariable regression analysis of the intervention impact comparing the study arms over time, children in the SP-AQ plus LNS area had significantly higher mean z-scores for MUAC than those in the SP-AQ only area. No difference in mean z-scores was observed between intervention arms with respect to any other nutritional anthropometric outcome (Table 3).

### Case-control study results

In the case-control study, 380 children were recruited, of whom, 368 children were included in the analysis (Table 1, Objective 3). In the SP-AQ arm, 156/179 recruited children received SP-AQ (87.2%); 3 children also received LNS and were excluded from analysis. In the SP-AQ plus LNS arm, 181/189 recruited children received SP-AQ plus LNS (95.8%); 9 children were excluded from analysis as they only received SP-AQ. None of the recruited children received only LNS. Of those tested for malaria in participating health facilities, 93 children (24.5%) tested positive and 275 (72.4%) tested negative. The adjusted odds of clinical malaria among children who reported taking SP-AQ within 30 days of consultation were 77% lower (OR = 0.23; 95% CI 0.09–0.6; p = 0.003) than among those who did not receive any intervention in the SP-AQ only area. The odds of clinical malaria among those who took SP-AQ plus within 30 days of consultation were 78% lower than among those who did not receive any intervention (OR = 0.22, 95% CI 0.09–0.55, p = 0.001). The interaction between site and intervention arm was not significant (OR = 0.98, 95% CI 0.19–5.05, p = 0.986) (Table 4).

**Table 4:** Characteristics and logistic regression results for malaria outcomes (positive mRDT) in recruited case-control study participants.

	Controls [n = 275] Mean% (95% CI)	Cases [n = 93] Mean% (95% CI)	p-value	Crude OR (95% CI)	Adjusted OR (95% CI)	p-value
**Age (months)**	15.7	16.9				
6-11m (n = 93)	25.1 (20.3–30.6)	25.8 (17.8–35.7)		1	1	
12-27m (n = 275)	74.9 (69.4–79.7)	74.2 (64.3–82.1)	0.891	0.96 (0.45–2.04)	1.35 (0.65–2.8)	0.42
**Gender**						
Male (n = 190)	50.9 (45.0–56.8)	53.8 (43.5–63.7)		1	1	
Female (n = 178)	49.1 (69.4–79.7)	46.2 (36.3–56.5)	0.63	0.89 (0.66–2.04)	0.96 (0.65–1.4)	0.82
**Intervention received**						
None (n = 31)	4.7 (2.8–8.0)	19.4 (12.5–28.7)		1	1	
SP-AQ or SP-AQ plus LNS (n = 337)	95.3 (92.0–97.2)	80.6 (71.3–87.5)	<0.001	0.21 (0.1–0.45)	0.23 (0.09–0.6)	0.003
**Site**						
SP-AQ only (n = 179)	42.5 (36.8–48.5)	66.7 (56.4–75.5)		1	1	
SP-AQ plus LNS (n = 189)	57.5 (51.5–63.2)	33.3 (24.5–43.6)	<0.001	0.37 (0.11–1.24)	0.39 (0.15–0.96)	0.04
Interaction Intervention * Site					0.98 (0.19–5.05)	0.99
**Presence of other symptoms**						
No (n = 207)	62.8 (56.7–68.4)	42.7 (32.8–53.2)		1	1	
Yes (n = 151)	37.2 (31.6–43.1)	57.3 (46.8–67.2)	0.001	2.27 (0.94–5.45)	2.28 (0.8–6.54)	0.12
**Bednet use in previous night**						
No (n = 207)	14.6 (10.8–19.3)	21.5 (14.3–31.1)		1	1	
Yes (n = 308)	85.5 (80.7–89.2)	78.5 (68.9–85.7)	0.116	0.62 (0.25–1.58)	0.85 (0.33–2.17)	0.74
**Indoor spraying in child’s household**						
No (n = 364)	99.3 (97.1–99.8)	100				
Yes (n = 2)	0.70 (0.18–2.90)	0	0.41	NA		
**Month of recruitment**						
August and September (n = 108)	29.5 (24.3–35.1)	29.0 (20.7–39.1)		1	1	
October (n = 131)	34.2 (28.8–40.0)	39.8 (30.3–50.1)		1.18 (0.65–2.14)	1.41 (0.65–3.1)	0.39
November (n = 129)	36.3 (30.9–42.2)	31.2 (22.5–41.4)	0.56	0.87 (0.36–2.12)	1.9 (0.37–2.68)	0.99

## Discussion

The results of this study indicated nearly 90% coverage was achieved through door-to-door delivery of the intervention. The high coverage attained during this intervention may be partly credited to the local knowledge and experience of the CDDs in Kano State, who also work on polio vaccination campaigns and other door-to-door delivery schemes for public health. This result is important to public health intervention programming because coverage was high despite the potential for decreased operational efficiency due to delivery of two interventions. The LNS product used in this study was packaged in cartons of 36 325-gram cups (a one-week supply per cup), requiring four cups delivered per eligible child each month [48]. Thus, each 11.7-kilogram carton covered nine children, making LNS a considerable additional weight to carry for CDDs who may visit remote areas with no motorized transportation. Moreover, approximately 80% of children who received SP-AQ reportedly completed the three-day course across intervention arms. These results suggest that bundling interventions did not negatively impact coverage or compliance in an SMC campaign with an existing cadre of CDDs.

This study also provides further evidence that SMC exposure is associated with reduced clinical malaria episodes, an important result to consider as SMC is scaled up across the Sahel, where reaching 23 million children considered at risk is a major operational challenge [27].

Apart from an improvement in MUAC z-scores, no impact was demonstrated on nutrition outcomes with the addition of LNS to the SMC campaign. While an impact on stunting and mean HAZ would not be expected due to the relatively brief timeframe of the intervention, some improvements in weight gain and reductions in wasting were expected within the SP-AQ plus LNS intervention arm. LNS may not have been consumed in the appropriate doses to generate significant impact, or the type of supplement selected may not have delivered sufficient nutrients for children at high risk for malnutrition, even when provided at the recommended dose. Other LNS evaluations have shown mixed results across intervention settings [28–31,49]. It is also possible that the significant changes in nutritional indicators between baseline and endline across intervention arms masked the impact of the LNS.

The differences in anthropometric indicators over time across both study arms were unanticipated. The very high prevalence of stunting at baseline and midline and the apparent marked improvement of both percent stunting and mean LAZ at endline was particularly striking. Also striking was that baseline and midline WLZ were not correspondingly low as would be expected in a very stunted, nutritionally compromised population. These observations may be explained by several factors, and it is likely that a mix of these reasons rather than one alone is responsible. First, there may have been seasonal improvements in food availability [50]–this is consistent with WAZ (and to a lesser extent mean MUAC) being better at endline than at baseline or midline. Similarly, it is also possible that the SP-AQ intervention contributed to improvements in endline anthropometry by reducing the frequency and/or severity of infections among participating children–this is also consistent with improvements in WAZ between survey rounds. Next, measurement error may have played a part–this is particularly true for length-based measures which account for the majority of our exclusions. Length is the most technically difficult measurement to do; for instance, the legs of young infants may not have been adequately extended at baseline and midline (legs are normally held in slight flexion, the younger the infant the greater that being). This would have led to falsely low length measurements and in turn falsely low LAZ and falsely high rates of stunting. This would also explain the apparently ‘normal’ WLZ: flexed legs lead to falsely high WLZ. Measurement errors could also have arisen due to our length boards having dual units (inches and centimeters) printed on the measurement tape. Finally, since we conducted repeat cross-sectional surveys rather than cohort follow-up of the same children, it is possible that just by chance a wealthier, healthier, better nourished cohort was selected at endline compared to baseline. This would explain Table 3: taking socioeconomic status into account makes most cross-time comparisons of anthropometry statistically non-significant. Regarding the impact of these issues on validity of our remaining/overall anthropometric data: we consider our endline data to be especially robust since there were very few exclusions at this time point; whilst different numbers of exclusions at different time points might affect the validity of cross-time comparisons, inter-group comparisons would not be affected. Moreover, not all the anthropometric measures were equally affected–any uncertainty about our length-based measures did not affect weight-based measures (WAZ) and MUAC, hence those should be seen as reliable indicators of changes in anthropometric status over time.

Frequent infections are known to contribute to poor nutritional status by increasing the body’s nutritional demands, reducing appetite, and driving direct nutrient losses [5,9]. Similar results have been observed with the use of trimethoprim-sulphamethoxazole (TS) prophylaxis among HIV-exposed children in rural Uganda, which resulted in reductions in rates of stunting [4]. However, in the absence of a control group not provided with SP-AQ, no clear conclusions on the impact of SP-AQ on nutritional outcomes can be made.

The odds of clinical malaria were lower in children who took SP-AQ compared to those who did not in this study, providing further evidence that exposure to SMC has a protective effect on clinical malaria incidence [26,27]. The addition of LNS did not modify the effect of SP-AQ in this study. Even though the relationship between malaria and malnutrition is not yet clear [19], an interaction remains biologically plausible as improvements in nutrition strengthen the immune system and make the body more capable of fighting off infection [28,32]. Larger, multi-center case-control studies are needed to confirm the observed findings that adding LNS to SMC campaign reduced the odds of getting clinical malaria in this and other settings.

### Generalizability and limitations

This study may provide useful considerations for communities in the Sahel sub-region of Africa where SMC campaigns are currently planned or ongoing. The cost-effectiveness of scaling up such a program must also be considered; purchasing LNS may be a substantial barrier as the cost of LNS per child, including shipping and taxes, is over 20 times the cost of SP-AQ and bundling SP-AQ with LNS added 18% to the cost of an SP-AQ-only delivery campaign.

This study was subject to several limitations. First, the repeated cross-sectional study design resulted in measurements taken from different children within the study population during each survey round. This made it possible to assess changes in nutritional measurements only at the population level. In addition, it was not possible to assess the potential impact of SP-AQ on nutrition without a comparison group for the LNS intervention.

Second, possible measurement errors resulted in considerable loss of data available for anthropometric analysis. Twenty-three percent of surveyed households at midline were excluded due to an error in the eligible age range of children during sampling, resulting in uneven sample sizes between survey rounds. As discussed, a number of children were also excluded from analysis due to extreme anthropometric z-scores (Fig 1); this could have been due to errors in carrying out and recording anthropometric measurements, especially length-based measures and especially during the baseline and midline surveys. Whilst this means that cross-time comparisons should be made with great care, it does not invalidate inter-group comparisons. The low percentage of exclusions at endline suggests that anthropometric data at this final, most important time point are robust. Non-length based measures, which also resulted in few exclusions, should also be seen as more robust and reliable than length-based indices.

Third, recall bias may have been introduced in the surveys, especially around dosage for Plumpy’Doz. The recommended dosage is 4.5 grams per day for children under one year of age and nine grams per day for children 1–2 years of age; reported quantities consumed ranged from 1–20 grams per day. This wide range of dosages may be reflective of problems with recall, sharing the commodity with other members of the household, or misunderstanding dosage instructions. Inconsistent dosing may have under- or over-estimated the overall effect of LNS following the intervention.

Fourth, baseline food availability was reportedly high, and remained high throughout the study, which may have limited our ability to detect an impact of adding LNS on health outcomes. However, while two-thirds of households reported no food insecurity at baseline, more than 70% of children measured at baseline were found to be stunted. This could indicate discordance between households’ perception of food security and actual nutrient intake by young children, or could point to other contributing factors, such as frequent infection and/or poor water, sanitation, and hygiene practices.

Finally, the nested case-control was subject to several limitations by design. Selection bias may have been introduced since only public health facility patients were included; children who sought treatment in the private sector, where nearly half of malaria treatment-seeking in Nigeria is estimated to occur, would not have had the opportunity to participate [51]. In addition, only patients presenting with febrile illness were enrolled; children with parastaemia presenting without fever could have been missed. Misclassification of positives and negatives was possible as well. Controls were children seeking care for illness, and the range of severity of these conditions or other, non-febrile health status, including malnutrition, is not known. Lastly, the case-control study results were subject to the same limitations as the cross-sectional surveys, including recall of exposure to SP-AQ and LNS and baseline food security.

### Conclusions

This study investigated the operational and health impacts of combining nutrition and antimalarial interventions. The sustained high coverage and adherence to an integrated intervention strategy supports the efficiency of such bundled campaigns, and provides evidence that SMC campaigns can be further improved beyond the current model. Whilst our anthropometric outcomes (especially length-based measures and the over-time comparisons) should be interpreted with care due to possible measurement and other limitations discussed, results of the case-control study indicated that the addition of nutrition interventions may be beneficial to reduction of malaria. The best nutrition products in terms of cost efficiency and health outcomes should be considered as candidates for distribution in malnourished areas across the Sahel, including other types of LNS and other WHO-recommended supplementary foods such as centrally-produced fortified blended foods or micronutrient powders. Alternatively, “nutrition-sensitive” interventions such as vouchers or cash, as was recently tested with successful outcomes in Niger [52,53], could be considered. Altering dosage and timelines for implementation, and the addition of educational interventions for caregivers should also be considered.

More generally, this study provides new evidence for how delivery of community health interventions may be improved. Given that addition of LNS, a bulky product, had no impact on coverage achieved through SMC campaigns, programs could consider adding different types of products to existing campaigns to enhance the efficiency of improving child health, such as vaccines, essential medicines, education, and other health interventions.

## Supporting information

S1 Fig Study area. Children in Kafin Agur, Burji, Kanwa, and Kauran Mata Wards received only SP-AQ (SP-AQ only area), while children in Kubaraci, Rikadawa, and Yakun received Plumpy’Doz LNS in addition to SP-AQ (SP-AQ plus LNS area).
(TIF)

S1 Table Probability proportional to size calculation. (DOCX)

S2 Table Detailed Characteristics of Sample Children and Households. Variables denoted with (*) were similar at baseline and endline but significantly different at midline (p<0.05).
(DOCX)

S3 Table Coverage of SP-AQ and LNS by number of doses received as measured during the midline survey (November 2014, immediately following the final distribution round) and the endline survey (May 2015). (DOCX)

S4 Table Nutritional outcomes of children sampled. (DOCX)

S1 File Trial Protocol. (PDF)

S2 File Case Control Register. (DOCX)

S3 File CONSORT Checklist. (DOCX)
