# Supplementary figures and images for: Seasonal malaria chemoprevention packaged with malnutrition prevention in northern Nigeria: A pragmatic trial (SMAMP study) with nested case-control
Source: PLoS One. 2019 Jan 25;14(1):e0210692. doi: 10.1371/journal.pone.0210692 (PMC6347255; doi:10.1371/journal.pone.0210692)

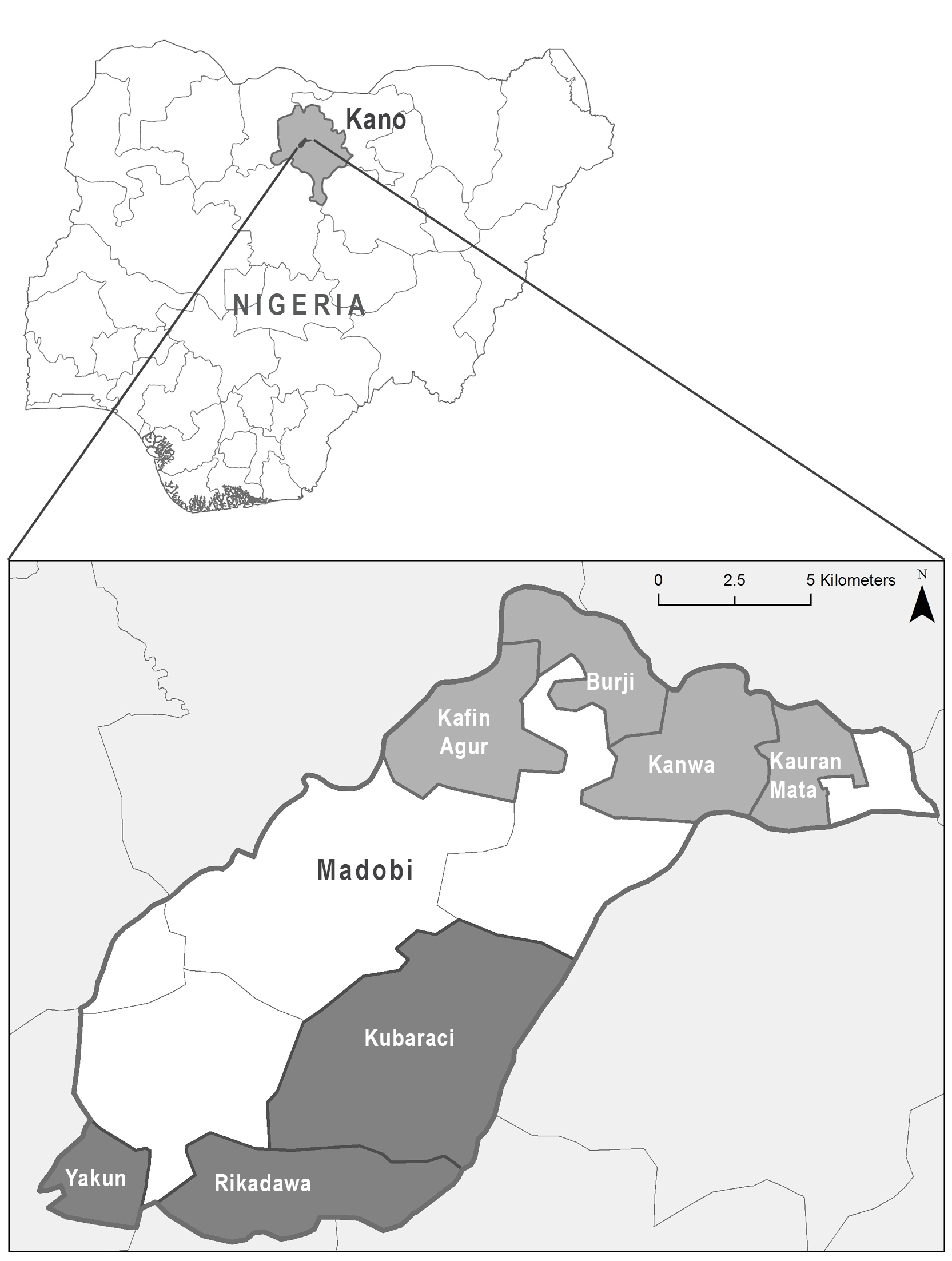

Supplement: S1 Fig — Children in Kafin Agur, Burji, Kanwa, and Kauran Mata Wards received only SP-AQ (SP-AQ only area), while children in Kubaraci, Rikadawa, and Yakun received Plumpy’Doz LNS in addition to SP-AQ (SP-AQ plus LNS area). (TIF) [file pone.0210692.s001.tif]
