# Supplementary material for: Seasonal malaria chemoprevention packaged with malnutrition prevention in northern Nigeria: A pragmatic trial (SMAMP study) with nested case-control
Source: PLoS One. 2019 Jan 25;14(1):e0210692. doi: 10.1371/journal.pone.0210692 (PMC6347255; doi:10.1371/journal.pone.0210692)
Supplement: S1 Table — (DOCX) [file pone.0210692.s002.docx]

| **S1 Table. Probablility Proportional to Size (PPS) EA Sampling per Ward** | | | | | | | |
| --- | --- | --- | --- | --- | --- | --- | --- |
| **Wards per Intervention Site** | **2014 U5 Population Projections by ward** | **Total EA maps per ward** | **Total no. of selected EA maps per LGA*** | **PPS of population by ward** | **Total No. of EAs by ward using PPS*** | **Actual No. of EAs by ward to enumerate using PPS**** | **No. household selected per ward**** |
| **SMC Sites** |  |  |  |  |  |  |  |
| Burji | 1,147 | 24 | 50 | 19.7% | 10 | 6 | 148 |
| Kafin Agur | 1,615 | 29 |  | 27.7% | 14 | 8 | 208 |
| Kanwa | 1,729 | 50 |  | 29.7% | 15 | 9 | 222 |
| Kauran Mata | 1,340 | 29 |  | 23.0% | 11 | 7 | 172 |
|  |  |  |  |  |  |  |  |
| **SMC & LNS Sites** |  |  |  |  |  |  |  |
| Kubaraci | 2,078 | 53 | 50 | 38.7% | 21 | 12 | 290 |
| Rikadawa | 2,478 | 45 |  | 46.1% | 24 | 14 | 346 |
| Yakun | 820 | 5 |  | 15.3% | 5 | 5 | 114 |
| **Total** | **11,207** | **235** | **100** |  | **100** | **60** | **1500** |
| * Including the 20 buffer EA maps per intervention area | | |  |  |  |  |  |
| * Assuming 30 EAs per intervention area | |  |  |  |  |  |  |
|  |  |  |  |  |  |  |  |
| ***Assumptions:*** |  |  |  |  |  |  |  |
| 25 HH per EA |  |  |  |  |  |  |  |
| 750 HH per intervention area | |  |  |  |  |  |  |
| 1,500 total HH |  |  |  |  |  |  |  |
