# Supplementary material for: Seasonal malaria chemoprevention packaged with malnutrition prevention in northern Nigeria: A pragmatic trial (SMAMP study) with nested case-control
Source: PLoS One. 2019 Jan 25;14(1):e0210692. doi: 10.1371/journal.pone.0210692 (PMC6347255; doi:10.1371/journal.pone.0210692)
Supplement: S2 Table — Variables denoted with (*) were similar at baseline and endline but significantly different at midline (p<0.05). (DOCX) [file pone.0210692.s003.docx]

**S2 Table** Detailed Characteristics of Sample Children and Households. Variables denoted with (*) were similar at baseline and endline but significantly different at midline (p<0.05).

|  | Baseline | | Midline | | Endline | |
| --- | --- | --- | --- | --- | --- | --- |
|  | *SP-AQ Only n=751* | *SP-AQ+LNS n=741* | *SP-AQ Only n=656* | *SP-AQ+LNS n=564* | *SP-AQ Only n=775* | *SP-AQ+LNS n=804* |
| Child age | 6-24 months | | 9-27 months | | 15-33 months | |
| Number of children | 860 | 840 | 650 | 803 | 861 | 954 |
| Respondent is Head of Household (proportion, 95% CI) | 68.0%  (56.9-79.1) | 73.4%  (65.6-81.4) | 69.5%  (62.3-76.7) | 69.3%  (61.9-76.7) | 70.8%  (64.4-77.2) | 69.7%  (62.2-77.2) |
| Respondent is Primary Caregiver (proportion, 95% CI) | 38.5%  (26.8-50.1) | 35%  (25.8-44.2) | 43%  (32.1-53.8) | 44.8%  (37.1-52.4) | 39.7%  (29.6-49.8) | 39.8%  (32.1-47.5) |
| Sex of Head of Household (proportion male, 95% CI) | 98.7%  (97.5-99.9) | 98.4%  (97.4-99.4) | 99.7%  (99.1-100) | 98.4%  (97.0-99.8) | 98.7%  (97.7-99.8) | 99.1%  (98.3-99.9) |
| Sex of Primary Caregiver (proportion male, 95% CI) | 42%  (23.1-60.9) | 48.8%  (33.0-64.7) | 52.8%  (38.7-67.0) | 46%  (35.7-56.2) | 53.8%  (42.9-64.6) | 44.7%  (31.6-57.7) |
| *Education* (proportion, 95% CI) |  |  |  |  |  |  |
| Primary or less (inc. Islamiyya/Quranic)* | 81%  (73.0-88.9) | 86.2%  (80.6-91.7) | 65%  (57.6-72.3) | 69.4%  (62.6-76.2) | 81.9%  (76.9-86.8) | 81.4%  (76.3-86.5) |
| Secondary or Higher | 19%  (11.1-27.0) | 13.8%  (8.3-19.4) | 35%*  (27.7-42.4) | 30.6%*  (23.8-37.4) | 18.1%  (13.2-23.1) | 18.6%  (13.5-23.7) |
| Religion – Islam (proportion) | 100% | 100% | 100% | 100% | 100% | 100% |
| *Ethnicity* (proportion, 95% CI) |  |  |  |  |  |  |
| Hausa | 98.2%  (95.2-100) | 98.4%  (96.9-99.8) | 97%  (94.1-100) | 96.1%  (92.0-100) | 98.4%  (97.1-99.6) | 95.2%  (91.4-99.1) |
| Fulani | 1.8%  (0.0-4.8) | 1.6 %  (0.2-3.1) | 3%  (<0.1-5.9) | 3.9%  (0.0-8.0) | 1.6%  (0.4-2.9) | 4.8%  (0.9-8.6) |
| Total Household Members (median, range) | - | - | 7  (2–20) | 7  (2–20) | 7  (3–20) | 7  (2–20) |
| Total LNS-eligible children in Household (median, range) | 1  (1-3) | 1  (1-4) | 1  (1-3) | 1  (1-4) | 1  (1-3) | 1  (1-5) |
| Primary Caregivers in Household (proportion with 1 caregiver, 95% CI)* | 73.4%  (67.8-79.0) | 72%  (63.3-80.8) | 49.8%  (43.2-56.3) | 51.2%  (44.2-58.1) | 83.1%  (74.9-87.8) | 73.1%  (67.2-78.9) |
| Wealth Index Quintiles (proportion, 95% CI, adjusted to baseline) | p=0.051 between sites | | p=0.018 between sites | | p=0.06 between sites | |
| Lowest | 16.8%  (8.5-25.2) | 22%  (13.4-30.6) | 10.9%  (6.2-15.7) | 20.7%  (12.9-28.5) | 14.5%  (8.8-20.2) | 22.9%  (15.8-29.8) |
| Lower | 16.3%  (10.8-21.8) | 24.4%  (20.0-28.9) | 14.8%  (10.7-18.8) | 18.7%  (13.9-23.5) | 16.4%  (10.1-22.6) | 16.3%  (12.2-20.4) |
| Middle | 17.6%  (13.6-21.6) | 22.1%  (17.5-26.6) | 26.3%  (21.8-30.7) | 25.1%  (20.5-30.0) | 20.1%  (15.3-24.9) | 24.2%  (19.4-29.0) |
| Higher | 23.3%  (18.0-28.5) | 16.5%  (12.3-20.7) | 20.9%  (17.4-24.4) | 17.6%  (12.0-23.1) | 23.1%  (17.8-28.4) | 20.1%  (14.1-26.0) |
| Highest | 26%  (17.1-34.9) | 14.9%  (8.6-21.3) | 27.7%  (21.0-34.3) | 18.3%  (12.3-24.2) | 25.9%  (16.6-35.3) | 17%  (11.1-22.9) |
| Household Self-Reported Food Secure | 65.7%  (54.0–75.7) | 66.5%  (59.5–72.8) | 64.6%  (55.9–72.5) | 68.2%  (59.1–76.1) | 64.1%  (54.4–72.8) | 61.2%  (52.0–69.5) |
