# Supplementary material for: Seasonal malaria chemoprevention packaged with malnutrition prevention in northern Nigeria: A pragmatic trial (SMAMP study) with nested case-control
Source: PLoS One. 2019 Jan 25;14(1):e0210692. doi: 10.1371/journal.pone.0210692 (PMC6347255; doi:10.1371/journal.pone.0210692)
Supplement: S3 Table — (DOCX) [file pone.0210692.s004.docx]

**S3 Table** Coverage of SP-AQ and LNS by number of doses received as measured during the midline survey (November 2014, immediately following the final distribution round) and the endline survey (May 2015).

| **Characteristic** | **Midline** | | | **Endline** | | |
| --- | --- | --- | --- | --- | --- | --- |
|  | **SP-AQ**  **[650]** | **SP-AQ+LNS [803]** | | **SP-AQ**  **[861]** | **SP-AQ+LNS [954]** | |
|  |  | **SP-AQ** | **LNS** |  | **SP-AQ** | **LNS** |
| Number of doses received  (proportion, 95% CI) | n=649 | n=803 | n=800 | n=859 | n=944 | n=954 |
| None | 5.6%  (2.4–12.4) | 1.2%  (0.5–2.5) | 2.3%  (1.2–4.5) | 1.2%  (0.4–3.3) | 1.0%  (0.4–2.4) | 1.0%  (0.3–2.2) |
| One | 2.6%  (1.0–6.6) | 0.1%  (0.0–0.2) | 2.9%  (1.2–6.8) | 1.1%  (0.2–5.1) | 2.9%  (0.5–15.0) | 2.7%  (0.5–12.9) |
| Two | 6.3%  (3.9–10.2) | 14.2%  (5.7–31.2) | 18.6%  (8.8–35.0) | 7.1%  (2.7–17.0) | 2.7%  (1.1–6.3) | 3.2%  (1.7–5.8) |
| Three | 38.2%  (28.7–49.0) | 16.6%  (11.4–23.5) | 25.6%  (18.2–34.9) | 7.1  (3.4–14.5) | 4.5%  (2.5–7.9) | 8.9%  (5.3–14.5) |
| Four | 47.1%  (36.4–58.1) | 67.4%  (54.6–78.0) | 50.5%  (37.4–63.6) | 83.6%  (70.4–91.6) | 88.9%  (81.4–93.7) | 84.1%  (73.1–91.1) |
