# Supplementary material for: Seasonal malaria chemoprevention packaged with malnutrition prevention in northern Nigeria: A pragmatic trial (SMAMP study) with nested case-control
Source: PLoS One. 2019 Jan 25;14(1):e0210692. doi: 10.1371/journal.pone.0210692 (PMC6347255; doi:10.1371/journal.pone.0210692)
Supplement: S4 Table — (DOCX) [file pone.0210692.s005.docx]

**S4 Table** Nutritional outcomes of children sampled.

| **Characteristic** | **Baseline** | | **Midline** | | **Endline** | |
| --- | --- | --- | --- | --- | --- | --- |
|  | **SP-AQ [860]** | **SP-AQ +LNS**  **[840]** | **SP-AQ [650]** | **SP-AQ +LNS**  **[803]** | **SP-AQ [861]** | **SP-AQ +LNS**  **[954]** |
| Length-for-age z-score  (mean, 95% CI, n) | -3.2  (-3.4–-2.9)  736 | -3.1  (-3.3–-2.8)  649 | -3.2  (-3.5–-3.0)  487 | -3.0  (-3.4–-2.7)  683 | -2.1  (-2.3–-2.0)  858 | -2.1  (-2.3–-1.9)  947 |
| Weight-for-length z-score  (mean, 95% CI, n) | -0.1  (-0.4–0.1)  785 | 0.0  (-0.3–0.3)  745 | 0.0  (-0.4–0.3)  522 | -0.2  (-0.6–0.1)  728 | -0.2  (-0.4–0.0)  858 | -0.2  (-0.5–0.0)  946 |
| Weight-for-age z-score  (mean, 95% CI, n) | -2.1  (-2.3–-1.9)  856 | -2.0  (-2.3–-1.7)  826 | -2.1  (-2.4–-1.8)  633 | -2.1  (-2.3–-1.9)  795 | -1.3  (-1.4–-1.1)  861 | -1.3  (-1.5–-1.0)  952 |
| MUAC z-score  (mean, 95% CI, n) | -1.0  (-1.2–-0.8)  860 | -1.2  (-1.4–-0.9)  840 | -1.1  (-1.3–-0.9)  643 | -1.0  (-1.2–-0.8)  797 | -1.0  (-1.1–-0.8)  861 | -0.6  (-0.7–-0.4)  951 |
| Stunting (HAZ <-2)  (proportion, 95% CI, n) | 78.9%  (73.0–83.8)  736 | 74.9%  (68.9–80.1)  649 | 77.9%  (71.3–83.4)  487 | 73.7%  (65.6–80.5)  683 | 53.8%  (48.7–58.8)  858 | 52.5%  (46.3–58.5)  947 |
| Wasting (WHZ <-2)  (proportion, 95% CI, n) | 15.1%  (11.9–18.9)  785 | 13.8%  (10.1–18.4)  745 | 18.2%  (13.8–23.6)  552 | 20.7  (15.0–27.8)  728 | 11.5%  (8.4–15.5)  858 | 15.9%  (11.4–21.8)  946 |
| Underweight (WAZ <-2)  (proportion, 95% CI, n) | 55.1%  (49.8–60.2)  856 | 51.5%  (43.5–59.5)  826 | 56.4%  (47.3–65.2)  633 | 52.0%  (44.5–59.4)  795 | 27.2%  (23.3–31.4)  861 | 31.2%  (24.2–39.2)  952 |
| Low MUAC  (proportion, 95% CI, n) | 3.8%  (2.0–7.0)  860 | 5.7%  (3.9–8.4)  840 | 0.1%  (0.0–0.7)  643 | 0.3%  (0.1–1.2)  797 | 0.6%  (0.2–1.8)  861 | 0.7%  (0.2–2.6)  954 |
| Severe acute malnutrition  (proportion, 95% CI, n) | 10.1%  (7.0–14.4)  860 | 11.6%  (8.5–15.6)  840 | 6.2%  (4.3–8.8)  587 | 10.3%  (7.3–14.6)  742 | 3.8%  (2.3–6.0)  858 | 4.8%  (2.9–7.8)  947 |
