# Supplementary material for: Seasonal malaria chemoprevention packaged with malnutrition prevention in northern Nigeria: A pragmatic trial (SMAMP study) with nested case-control
Source: PLoS One. 2019 Jan 25;14(1):e0210692. doi: 10.1371/journal.pone.0210692 (PMC6347255; doi:10.1371/journal.pone.0210692)
Supplement: S1 File — (PDF) [file pone.0210692.s006.pdf]

Testing packaging the Delivery of a Nutrition Intervention through the Seasonal Malaria Chemoprevention Campaigns on Coverage and Health Impact among Children in Kano State, Northern Nigeria.

Research Protocol

Agency: Clinton Health Access Initiative

Investigators: Owowunmi Omoniwa, Justin Graves, Karen Milch, Antoinette Bhattacharya, Leslie Emegbuonye, Arnuad Le Menach, Abigail Ward, Kathleen Maloney, Owens Wiwa, Kabir Masokano

Principal Investigator: Owens Wiwa

Acronyms

SAM	-	Severe Acute Malnutrition
CHAI	-	Clinton Health Access Initiative
KSMOH	-	Kano State Ministry of Health
SMC	-	Seasonal Malaria Chemoprevention
WHO	-	World Health Organization
SP - AQ	-	Sulfadoxine-pyrimethamine + Amodiaquine
KSMCP	-	Kano State Malaria Control Program
LNS	-	Lipid-based Nutritional Supplement
Q-1	-	Question 1
Q-2	-	Question 2
Q-3	-	Question 3
H-1	-	Hypothesis 1
H-2	-	Hypothesis 2
H-3	-	Hypothesis 3
LGA	-	Local Government Area
BCC	-	Behavioral Change Campaign
CDD	-	Community Drug Distributor
ID	-	Identification number
DOT	-	Directly Observed Therapy
SAM	-	Severe Acute Malnutrition
HIV	-	Human Immunodeficiency Virus
EA	-	Enumeration Area
FGD	-	Focus Group Discussion
RDT	-	Rapid Diagnostic Test
DHOs	-	District Health Officials
SP	-	Sulfadoxine-pyrimethamine
AQ	-	Amodiaquine
HIV	-	Human Immunodeficiency Virus
ARF	-	Adverse Reaction Form
IRB	-	Institutional Review Board

Table of Contents

1 Introduction ... 4

2 Research Goal, Objectives, and Significance ... 5

3 Hypotheses and Success Metrics ... 6

4 Intervention Design ... 9

  Study Site and Population ... 9

  Overview of the Intervention Arms ... 9

    Phase 1: Community Sensitization ... 9

    Phase 2: Intervention Administration ... 10

    SP-AQ Administration ... 11

    Dosing Procedures ... 11

    Inclusion/Exclusion Criteria ... 11

5 Monitoring and Evaluation Methods ... 14

6 Ethical Considerations ... 18

  Potential Risks to Participants ... 19

  Potential Benefits to Participants ... 20

  Informed Consent and Participant Rights ... 20

  Data Management and Confidentiality ... 20

7 Dissemination Plan ... 21

8 Potential for Policy Impact ... 21

1 Introduction

In 2010, malaria claimed an estimated 660,000 lives globally, with 90% of these deaths occurring among children under-five and pregnant women. Nigeria alone represents more than one-quarter of all global malaria deaths[1] and has a population of over 9 million children under-five years of age in the northern States. Malnutrition is the number one risk factor for under-five child deaths and is an underlying factor in the death of an approximately 400,000 children in Nigeria each year.[2] Previous studies suggest there is an association between malaria and nutrition. Nearly 50% of all malaria deaths are attributable to malnutrition.[3] Investment in malaria interventions has been shown to significantly increase mean weight-for-age and mid-upper arm circumference scores which are both recognized nutrition indicators.[4,5,6] Furthermore, malnutrition can increase the severity of malaria.[7] Nigeria has the highest prevalence of malnourished children in all of Africa, with nearly 9% of children under the age of five (approximately 2.4 million) suffering from severe acute malnutrition (SAM). In several of Nigeria's northern states, the levels of acute malnutrition exceed the WHO "emergency" threshold of 15%.[8]

Nigeria's northern State of Kano, has a population of nearly 2.5 million children under-five and an under-five mortality rate of 217 per 1,000.[9] Twenty-five percent of all under-five deaths in Kano (approximately 132,000 annually) are attributable to malaria.[10] To address this burden, in 2013, the Clinton Health Access Initiative (CHAI) in collaboration with the Kano State Ministry of Health (KSMOH) in Northern Nigeria piloted the delivery of Seasonal Malaria Chemoprevention (SMC) as an approach to reduce the burden of malaria. SMC was endorsed by the World Health Organization (WHO) in March 2012 and is defined as the intermittent administration of a full treatment course of antimalarial medication to children living in areas of highly seasonal transmission. The purpose of SMC is to prevent malaria by maintaining therapeutic antimalarial drug concentrations in the bloodstream throughout the period of greatest malarial risk. The WHO recommends SMC for children under-five years living in the Sahel sub-region of Africa during the period known as the "long rains."[8] One recommended antimalarial dosing structure for SMC is four treatment courses of sulfadoxine-pyrimethamine + amodiaquine (SP-AQ), offered as a three day dosing regimen, taken once per month for four consecutive months over the

---

[1] *World Malaria Report 2012*. Geneva: World Health Organization, 2012. Internet resource.
[2] Calculated by applying the 45% of all child deaths attributable to malnutrition to the 3.24 million child deaths in sub-Saharan Africa reported in the Levels & Trends in Child Mortality Report from UNICEF, WHO, the World Bank, and the UN in 2013.
[3] The Lancet. Maternal and Child Undernutrition: Global and Regional Exposures and Health Consequences. January, 2008.
[4] Snow R, Molyneux C, Njeru E, et al. 1997. The effects of malaria control on nutritional status in infancy. Acta Tropica 65: 1–10.
[5] Ntab B. Cisse B, Boulanger D, Sokhna C, et al. 2007. Impact of intermittent preventive anti-malarial treatment on the growth and nutritional status of pre-school children in rural Senegal. Am J Trop Med Hyg. 77(3): 411-417.
[6] Friedman, JF, Kwena AM, Mirel LB, Kariuki SK, . et al. 2005. Malaria and nutritional status among pre-school children: results from cross-sectional surveys in western Kenya. Am. J. Trop. Med. Hyg. 73(4): 698-704.
[7] Friedman, JF, Kwena AM, Mirel LB, Kariuki SK,.et al. 2005. Malaria and nutritional status among pre-school children: results from cross-sectional surveys in western Kenya. Am. J. Trop. Med. Hyg. 73(4): 698-704
[8] WHO: Global Database on Child Growth and Malnutrition. Severe Acute Malnutrition: http://www.who.int/nutgrowthdb/about/introduction/en/index5.html [Accessed July 12, 2014].
[9] Partners in International health. 2014. Maternal newborn and child health in Northern Nigeria. http://www.healthpartners-int.co.uk/our_projects/documents/PRRINN-MNCHProgrammeBrief.pdf [Accessed July 12, 2014].
[10] Nigerian National Population Council cites the 2014 population of under-fives in Kano State as 2,433,357.
[8] WHO Global Malaria Programme (March 2012). WHO Policy Recommendation: Seasonal Malaria Chemoprevention (SMC) for Plasmodium falciparum malaria control in highly seasonal transmission areas of the Sahel sub-region in Africa.

duration of the long rainy season.[9] The WHO recommendation results from evidence from successful field studies that show that SMC prevents approximately 75% of all malaria episodes and 75% of severe malaria episodes.[11]

In Kano State where CHAI has historically assisted the Kano State Malaria Control Program (KSMCP) with SMC delivery, malaria is highly seasonal and spikes during the rainy season, typically during the two to four month period beginning in July/August of each year when SMC is recommended. The rainy season coincides with the "hunger season", typically starting one month after the rains have begun. During these months children are extremely vulnerable to dying of causes that are completely preventable. One recommended treatment for malnutrition is Lipid-based Nutritional Supplements (LNS). Plumpy'Doz™ is LNS designed for the treatment of moderate acute malnutrition and the prevention of undernutrition. Plumpy'Doz™ is designed to sustain the growth of young children, improve their motor and cognitive development, and reduce the incidence of malnutrition—whether acute or chronic—during critical periods of food and nutrition insecurity. As they can be given without a medical prescription, LNS are often used in association with blanket feeding programs targeting young children who are not malnourished but who are at risk of malnutrition.

CHAI's 2013 SMC pilot demonstrated that SMC coverage was optimized through door-to-door delivery of SP-AQ where nearly 90% of eligible children received at least one treatment course of SMC during the intervention period. As the 2014 SMC campaign will rely on the door-to-door delivery method, using the 2014 SMC campaign to deliver LNS could be one avenue for reaching children at-risk for both malaria and malnutrition at a critical time for both health concerns; however, to date this has never been done. Beyond reductions in malaria and malnutrition, packaging LNS with the SMC campaign may serve as an added incentive for parents/guardians to accept SMC preventative treatment, adhere to dosing guidelines, and incentivize caregivers to stay at home during regularly scheduled delivery periods resulting higher SMC coverage. CHAI, in collaboration with the KSMOH, will test SMC and LNS coverage, malaria, and malnutrition outcomes reached by packaging the delivery of LNS through the door-to-door SMC campaign.

2 Research Goal, Objectives, and Significance

The primary goal of this study is **to compare the intervention coverage and health outcomes achieved among children 6-24 months by packaging the delivery of LNS through the SMC campaign as compared to the delivery of SMC alone.** This goal will be guided by three research questions presented in Table 1.

Table 1: Research questions.

[9] Sahelian sub-regions recommended for SMC are those with a clinical attack rate greater than 0.1 per transmission season in the target age group, or areas with >10 of 100 under-fives experiencing clinical malaria during the rainy season.
[11] WHO Global Malaria Programme (March 2012). *WHO Policy Recommendation: Seasonal Malaria Chemoprevention (SMC) for Plasmodium falciparum malaria control in highly seasonal transmission areas of the Sahel sub-region in Africa.*

#	Research Questions	Methodology
**Q-1**	Does packaging the delivery of LNS through the SMC campaign result in higher SMC coverage and adherence within target communities among children 6 to 24 months, compared to delivery of SMC alone?	Cross-sectional household survey (midline)
**Q-2**	Does packaging the delivery of LNS through SMC campaigns result in reduced malnutrition outcomes (stunting, wasting, and underweight) among children 6 to 24 months, compared to SMC alone?	Repeated cross-sectional household surveys (baseline, midline, and endline 6 months after the final round of interventions)
**Q-3**	Does treatment with SMC or the packaging of LNS through the SMC campaigns change the odds that a child is diagnosed with clinical malaria cases including severe cases among children 6 to 24 months, compared to SMC alone?	Malaria Case Tracker (case-control study)

The findings from this study will allow the Ministry of Health and State Malaria Control Programs in the nine eligible States for SMC in Nigeria to make a decision regarding whether LNS should be delivered through the SMC door-to-door campaigns in at risk settings in order to achieve high coverage rates and improve health outcomes. Additionally, the results will help inform global policy makers and implementers regarding the co-packaging of SMC and nutrition interventions.

3 Hypotheses and Success Metrics

The primary hypotheses that will be tested in this study as well as the endpoint indicators for success are listed in Tables 2 and 3 below and are based on three primary outcomes: coverage, reduction in malnutrition, and reduction in clinical malaria.

Table 2: Research Hypotheses

#	Hypotheses
**H-1**	Coverage and adherence of SMC among eligible children in areas where SMC + LNS was delivered will not differ from areas where only SMC was delivered.
**H-2**	Nutrition indicators among eligible children in areas where SMC + LNS was delivered will not differ from areas where only SMC was delivered.
**H-3**	The odds of being diagnosed with clinical malaria cases including severe cases among eligible children who received SMC + LNS, SMC, or neither treatment.

Table 3: Performance Metrics

**Hypotheses**	**Performance Indicator**	**Indicator Definition and Unit of Measurement**	**Data Source**
H-1	Monthly Coverage	*Monthly Coverage*<br><br>**Indicator Definition:** Percent of children 6-24 months* who received a dose of treatment^ out of the total number of eligible children surveyed during each administration period (July, August, Sept, and October).<br><br>**Unit of Measurement:** Percentage<br>* The age class will evolve and not always be 6-24 months.<br>^ Treatment refers to the intervention (either LNS + SMC or SMC alone).	Midline household survey
H-1	Adherence	*Adherence*<br><br>**Indicator Definition:** Percent of children 6-24 months who completed the three-day treatment course of SMC out of the total number of children who received SMC during the final round of intervention administration.<br><br>**Unit of Measurement:** Percentage<br>* The age class will evolve and not always be 6-24 months.	Midline Household survey
H-2	Malnutrition	*Stunting (Height for Age)*<br><br>**Indicator Definition:** Percent of children 6-24 months* whose height is more than 2 standard deviations below the median for the international reference population age 6-24 months. For children under 2 years recumbent length will be used.<br><br>**Unit of Measurement:** Percentage<br>* The age class will evolve and not always be 6-24 months.	Household surveys (baseline, midline, endline)
H-2		*Wasting (Weight for Height)*<br><br>**Indicator Definition:** Percent of children 6-24 months* whose weight for height is more than 2 standard	Household surveys (baseline, midline, endline)

		deviations below the median for the international reference population age 6-24 months. For children under 2 years recumbent length will be used.<br><br>**Unit of Measurement:** Percentage<br><br>* The age class will evolve and not always be 6-24 months.	
H-2		*(Underweight) (Weight for Age)*<br><br>**Indicator Definition:** Percent of children 6-24 months* whose weight for age is more than 2 standard deviations below the median for the international reference population age 6-24 months.<br><br>**Unit of Measurement:** Percentage<br><br>* The age class will evolve and not always be 6-24 months.	Household surveys (baseline, midline, endline)
H-3	Clinical Malaria	*Clinical Malaria*<br><br>**Indicator Definition:** Number of clinical confirmed malaria cases among children 6-24 months per 1,000 children 6-24 months in the estimated catchment areas of the facilities)<br><br>**Unit of Measurement:** Odds-Ratio among cases and controls, and incidence<br><br>* The age class will evolve and not always be 6-24 months.	Malaria case tracker(Case-Control study)
		Severe Malaria<br><br>**Indicator Definition:** Number of hospital admissions for confirmed severe malaria among children 6-24 months per 1,000 children 6-24 months in the estimated catchment areas of the facilities)<br><br>**Unit of Measurement:** Odds-Ratio among cases and controls, and incidence<br><br>* The age class will evolve and not always be 6-24 months.	Malaria case tracker (Case-Control study)

4 Intervention Design

Study Site and Population

Table 4: Overview of the study population for each treatment type.

Intervention Arm	LGA
Arm 1: SMC (Children 3-59 months)	Madobi
Arm 2: SMC (Children 3-59 months) + LNS (Children 6-24 months)	Madobi

This study has two intervention arms: SMC delivery and SMC + LNS delivery. Of note, SMC is specified by the WHO for children 3-59 months[12] and Plumpy'doz' is specified by UNICEF and WFP for children 6-24 months, this study will treat children in intervention arms based on these recommendations (see Table 4: Intervention Arm). In the Local Government Area (LGA), Madobi, between 1 and 3 wards were selected for each intervention arm. An overview of the study population for each intervention area is presented in Table 4. The intervention areas were chosen to be as similar as possible in terms of pre-determined criteria that included malaria incidence reported at public health centers, wasting prevalence, malaria endemicity, socio-economic status, urbanicity, mobility and cultural identity of the population, density of health facilities, presence of a CMAM facility, and population demographic structures (dependency ratio, fertility rate and household size, infant mortality, and birth spacing).

Overview of the Intervention Arms

Roll-out of the intervention will consist of two phases which are detailed below.

Phase 1: Community Sensitization

Sensitization to the intervention (either SMC or SMC + LNS) and the delivery method (door-to-door distribution) for each intervention arm area will take place on the community level using the pre-existing political structure and social network groups. Sensitization and mobilization will start at the state level with high-level advocacy meetings with key directorate heads in the KSMOH, head of hospitals management board, primary health care management board, and development partners. The SMC technical working group inaugurated in 2013 will gather to discuss progress, identify challenges, and

[12] WHO Gobal Malaria Programme (March 2012). WHO Policy Recommendation: Seasonal Malaria Chemoprevention (SMC) for Plasmodium falciparum malaria control in highly seasonal transmission areas of the Sahel sub-region in Africa.

develop mitigation strategies. Community level mobilization will then commence four weeks to the start of proposed administration period. In each community CHAI will support the KSMCP to host LGA and ward level meetings. Meetings will take place at the LGA secretariat or district/ward head's house and will bring together staff of the LGA Health Department and Health Education Unit as well as District, Ward, and village heads, religious leaders, and heads of health facilities. Participants will be sensitized to the treatments, benefits and risks, eligibility requirements, intervention timing, and procedures. Poster and fliers that have been created by the Behavioural Change Campaign (BCC)/Advocacy Unit of the KSMOH will be presented and then distributed to key stakeholders to be posted in health facilities and strategic places within their communities and for distribution within the community. Immediately after the meeting, village leaders will be supported to commence sensitization in their respective villages and ensure community uptake. One week prior to each administration period, ward, village, and health facility heads and religious leaders will announce intervention delivery details on a continuous basis to their catchment populations to reinforce the message. Additionally, town criers will be engaged to disseminate the message of treatment delivery. Of note, all parents/guardians will be requested to provide the drinking water for their child to ingest the drugs.

Phase 2: Intervention Administration

Phase 2 will make use of the existing network of Community Drug Dispensers (CDDs) that work on other health campaigns in Kano. Community lists will be obtained for each ward from the Village head or the CDD LGA Focal person. Within each ward, CHAI study staff will visit each identified community and a household sampling frame will be obtained from each community headman. In the event that a Village head cannot produce an accurate listing or new communities have developed that are not present on the village head's list, the village headman and CDDs will be used to create a census of their community. CDDs alongside study staff will visit each household on the sampling frame and obtain accurate listings of homes with at least one child under-five residing within it in the treatment area. Supplementary data will also be collected from parents/guardians including data on the number of children 3-59 months of age residing in each home, child and parent's/guardian's names, and contact details (e.g. phone number of parent/guardian, address of home).

CHAI will train pre-existing CDDs on the proper administration of SP-AQ/*Plumpy doz'* in a 1-day training course. During each administration period, each CDD will be assigned to 100 households that they will cover over the course of one week. The CDDs will visit children and their parents/guardians in their homes four times on a monthly basis (July-October) and deliver the treatment. After each visit, CDDs will instruct parents/guardians that they will return four weeks later, on the *same day of the week* as the first SMC visit to deliver the next treatment.

All treatments provided will be recorded in two places: a treatment card that is left with the parent/guardian of the child and the CDDs administration register. Each child will be assigned a unique Identification number (ID) and the treatment card and the child's name in the CDDs administration register will be labeled with the unique ID. The treatment card will be filled out by the CDD at first contact, and given to the parent/guardian of each child. Information collected on the treatment card will include the child's name, birthday, dose of SP-AQ and amount of LNS provided, date of

administration, and date of next visit. In addition to the information included on the treatment card, the CDD's administration register will also include the child's sex, parent's/guardian's name and the village's name and compound's address where the child resides. Children that meet the inclusion criteria in terms of age but do not meet all the inclusion criteria listed on pages 11-14 will be given a treatment card with a reference indicating why they were excluded from treatment this round. Each child's identity will be checked before treatment is given and no treatment will be provided without the parent's/guardian's written consent.

SP-AQ Administration

Dosing Procedures

The KSMOH and the KSMCP provided administration clearance for mass drug administration, dosing, and the study procedures. Dosing guidelines for SP-AQ are presented in Table 5 below and follow the WHO guidelines for SP-AQ delivery. SP-AQ will be provided once per month, ideally on the same day each month, for 4 consecutive months during the long rainy season (July 15 – October 15). A person trained in SP-AQ administration and the signs and symptoms of adverse reactions will administer the SP-AQ. Following drug ingestion, children will remain under observation for 15 minutes. In the event of spit-up or vomiting drugs will be re-administered alongside food, water, or breast milk. The first dose of SP-AQ, consisting of both SP and AQ tablets, will be administered through directly observed therapy (DOT) whereby children are observed taking the drug by the health worker or parent /guardian administering the drug. Parents/guardians will then be given the remainder of the co-blister (containing two AQ tablets each given on the respective two days) and instructions on how to administer the drug on days 2 and 3. In young children unable to swallow the SP-AQ, the CDD will recommend that the SP-AQ tablets be crushed and mixed with water and sugar. The CDD will provide sugar for the full three days of treatment.

Inclusion/Exclusion Criteria

All children 3-59 months at the first round of administration (July), residing in either intervention areas, and do not meet one of the exclusion criteria listed below are eligible to receive SMC. The child's parent/guardian must also sign an informed consent either with their signature or right thumbprint prior to administration.

Of note, if a child ages out during the study (is eligible at administration round 1 in July and subsequently turns 60 months prior to administration round 2, 3, or 4), he/she will remain enrolled in the intervention and continue to be eligible for SMC provided he/she does not meets the exclusion criteria below.

Exclusion Criteria:

- A child is 0-3 months of age or above 59 months of age.
- A child with severe acute illness or unable to take oral medication
- A child that has confirmed or suspected malaria. Confirmed malaria is defined as fever (body temperature>= 37.5 ° C) or history of fever in the last 24 hours and parasitologically positive by Rapid Diagnostic Test (RDT) or microscopy; in the

absence of RDT and microscopy diagnosis, malaria diagnosis should be based on clinical signs and symptoms.

- An HIV-positive child and receiving co-trimoxazole.
- A child who has received a dose of either AQ or SP drug more recently than the past month.
- A child who is allergic to either drug (AQ or SP).

Children with severe acute illness or high fever that are not currently under a clinician's care will be referred to the nearest public health facility.

Table 5: Dosing guidelines for eligible children.[9]

**Product and Dosing Information for SPAQ-CO**							
	Brand Name and Formulation: **SPAQ-CO**<br>Co-blister of Sulfadoxine/pyrimethamine tablets + Amodiaquine tablets (SP+AQ)<br>*Vendor*: Guilin Pharmaceuticals<br>*Catalog ID*: 3.2.05.28.001.01.02						
		Day 1		***Day 2***		***Day 3***	
**Product Name and Formulation**	**Age range**	**SP***	**AQ***	**SP***	**AQ***	**SP***	**AQ***
Infant Dose – AQ75mg + SP262.5mg	3 months to <12 months	1 tablet (250mg /12.5mg)	1 tablet<br>(75 mg)	**Nil**	1 tablet (75mg)	**Nil**	1 tablet<br>(75 mg)
Child dose – AQ150mg + SP525mg	12 months to 59 months	1 tablet<br>(500mg /25mg)	1 tablet<br>(150 mg)	**Nil**	1 tablet (150 mg)	**Nil**	1 tablet (150 mg)

***SP**: Sulfadoxine/Pyrimethamine tablets; AQ: Amodiaquine tablets

[9] *Guilin Pharmaceutical Co., Ltd.*

Plumpy'Doz' Administration

Dosing Procedures

For children in the SMC + LNS intervention arm, LNS will be provided to eligible children 6 to 24 months of age and is intended for daily use during the four-month intervention period. The monthly ration of LNS will be provided to the primary caregiver by the CDD at the same time of SP-AQ distribution. The first serving of LNS will be provided alongside the first dose of SP-AQ under DOT. The product does not require any preparation, and should not be cooked or diluted.[11] Caregivers will be instructed that LNS should be given in addition to breast milk and other available foods.[10] The LNS will not be provided to children less than 6 months of age, so as not to interfere with exclusive breastfeeding. Following the first dose, parents/guardians will be instructed to continue to serve eligible children LNS daily following the serving guidelines in Table 6. The recommended daily dose of Plumpy'Doz™ is 1.5 teaspoons (6-11 months of age) or 3 teaspoons (12-23 months of age), served 3 times per day; this is a total of 23 grams per day for children 6-11 months of age and 46 grams per day for children 12-23 months of age. One container of 325 grams is intended to serve one child for one week and each child will receive 4 containers for the entire month alongside the monthly SP-AQ treatment course. The pot will need to be resealed and stored in hygienic conditions between servings.

Table 6. Recommended Daily and Monthly Dosing of Plumpy'Doz

Age (months)	Recommended Plumpy'Doz Servings, per Day	Kilocalories, per Day	Number of Servings, per Day	Recommended Plumpy'Doz Servings, per Month
6-11	23 grams	123 kcal	3 servings	690 grams
12-23	46 grams	147 kcal	3 servings	1,380 grams

Inclusion/Exclusion Criteria

All children 6-24 months of age at the first round of administration (July), residing in the SMC + LNS intervention area, who do not meet one of the exclusion criteria listed below are eligible to receive LNS in addition to the SMC they will already be provided. The child's parent/guardian must also sign an informed consent either with their signature or right thumbprint prior to administration.

Of note, if a child ages out or in during the study (is eligible at administration round 1 in July and subsequently turns 25 months prior to administration round 2, 3, or 4), he/she will remain enrolled in the intervention and continue to be eligible for LNS provided he/she does not meet the exclusion criteria below. Also, if a child is below 6 months at administration round 1 but turns 6 months prior to administration round 2, 3, or 4 are eligible to be enrolled in rounds that occur after their 6 month birthday.

Exclusion Criteria:

[11] Supplementary Spread Product Details, UNICEF Supply Catalogue. Accessed June 2, 2014 at https://supply.unicef.org/.

- A child is 6-24 months of age.
- Any child diagnosed with severe acute malnutrition (SAM) will be excluded from the study and referred instead for treatment at the CMAM center.
- Any child with an identified allergy to peanuts will be excluded from participation (*Note: peanut allergies are rare in children under 2 years of age*).

5 Monitoring and Evaluation Methods

Table 7 presents the data collection methods used for evaluation of the performance metrics previously outlined in Table 3, and the target population. Estimating intervention treatment coverage will be assessed through the midline cross-sectional household survey. The impact of interventions on nutrition health outcomes will be assessed through the three repeated cross-sectional household surveys. The impact of interventions on malaria health outcomes will be assessed through a case-control study using a malaria tracker where cases will be recruited from health facilities and control from the midline household survey. Cases will be recruited from facilities due to the challenge of identifying sufficient numbers of acute malaria episodes via monthly household surveys. Focus Group Discussions (FGDs) will be used to provide context to the results. CHAI will contract a private data collection firm to carry out the household surveys, case control study, collection of health facility records, and completion of FGDs. CHAI will carry out the data analysis and provide CIFF the full clean datasets from the firm upon request to validate findings.

Table 7: The data collection methods used for evaluation of the performance metrics previously outlined in Table 3, and the target population.

Data Collection Method	Timeline	Inclusion Age*	Location	Indicators
Baseline Household Survey	July 2014	6-24 months	Community	Malnutrition
Midline Household Survey<br>Nutrition Module<br>SMC Coverage Module	November 2014	6-28 months<br>(3-63 months)^	Community	Malnutrition<br>Treatment Coverage
Endline Household Survey	May 2015	12-34 months	Community	Malnutrition Prevalence
Malaria Case Tracker	Continuous	6-24 months in July 2014	Health Facility<br>Community	Malaria Incidence
Focus Group Discussions	November 2014	Parents/Guardians/CDDs	Community	Qualitative data

*Note: Once a child in enrolled in the intervention, he/she will remain eligible even if he/she ages out during the study. Therefore, eligible age class continuously evolves.

^At midline the exclusion criteria is having a child 6-28 months of age, however, to estimate coverage among older children SMC coverage will be collected about all children 3-59 months of age living in the home.

Evaluation Data Collection Procedures and Participant Recruitment

Household Surveys

In total, three household surveys will be conducted, a baseline in July of 2014 alongside the first administration, midline in November 2014 after Round 4 of administration, and a final survey in May 2015, six months post intervention. The baseline survey will provide baseline information on demographics, and nutrition indicators. The midline survey will provide an estimate of the intervention coverage and adherence to SMC in addition to the nutrition indicators. The final survey six months after the endline administration will determine if there is a long-term health impact in terms of the nutrition indicators. Each questionnaire will be a pre-tested, interviewer-administered questionnaire lasting about 45-60 minutes.

For each survey, 1,500 households with at least one child that meets the inclusion age outlined in Table 6 will be selected to participate in the survey (a minimum of 750 households in each intervention study arm: SMC and SMC + LNS). This sample size is powered to measure a 10% difference in the outcome indicator (intervention coverage or nutrition indicator is estimated at 50%, which is conservative and gives the largest sample size) between the two intervention areas with an alpha of 5% and a power of 90%, and a design effect of 1.5.

A multistage stratified sampling strategy will be used for household selection. The sampling frame for the survey will come from the national sampling frame, which lists all the Enumeration Area (EA) maps of the 2006 Population and Housing Census conducted by the National Population Commission. Assuming 25[13] households are interviewed per EA, EAs are needed to achieve the total target sample size – in other words 30 EAs in each intervention arm. To account for the unlikely event that a randomly chosen EA did not contain 25 eligible households, 20 additional EAs will be randomly selected per intervention arm, making a total of 100 EAs selected per survey round (50 EAs per intervention arm). The supplemental EAs will only be used if needed. Using the 2013 population projections of each ward, we will use a probability-proportional-to-size methodology to select an appropriate number of EAs within each ward.

At the second stage, a systematic random sampling will be used to select 25 households per EA. The random selection of households in each EA will proceed as follows: starting from any point, the fieldworker will walk around the selected EA to identify and list all households. In listing the households, the address and the name of head of households will be recorded for only those households with at least one child that meets the inclusion age outlined in Table 7. All institutional households will be excluded. The number of eligible households in each EA will be divided by 25 to obtain the sampling interval k. A number j between 1 and k will be randomly selected using simple random sampling. The household corresponding to this number in the list of households will be the first household to be

[13] Should be possible to obtain desired sample size based on household demographic breakdown from CHAI's SMC 2013 household survey in Kano, Nigeria.

included in the sample. To determine subsequent households to be included in the sample, k will be added each time, i.e., j +k, j + 2k, j + 3k, etc., and k will vary from one EA to the other depending on the number of eligible households.

Any household within the study's catchment area with at least one child that meets the inclusion age outlined in Table 7 is eligible for selection. In selected households, an enumerator will visit the home and make an accurate listing of all children that meets the inclusion age outlined in Table 6. In each selected home, after the informed consent/assent is gathered, the head of household (either male or female) will be interviewed about the household demographics and caregivers will be asked specific questions about all eligible children in their care.

Household survey inclusion criteria are that the respondent must:

1.) Reside in the intervention catchment area.
2.) Be the primary parent/guardian or parent for a child that meets the inclusion age outlined in Table 7.
3.) Must provide informed consent (for parents/guardians who are between the ages of 12-18 they must provide informed assent and if their parent/guardian is nearby, this parent/guardian must also provide informed consent form).

Malaria Case Tracker

In order to assess the relationship between the treatments and malaria health outcomes, a case-control study will be used. The study population includes any child between the ages 6-24 months in July 2014 residing in the interventions areas. The study will begin following the first round of administration, and will continue until adequate numbers of cases and controls have been recruited (with a maximum of two months following the final round of distribution even though the estimated sample size may not be reached). The outcome of interest is confirmed clinical malaria (including severe cases) with cases defined as any child from the study population with a positive malaria test by either a Rapid Diagnostic Test (RDT) or microscopy. Controls are defined as children whose caregivers report they meet the following two inclusion criteria: 1.) no previous malaria symptoms (no fever in the last 4-weeks) and 2.) no care was sought for fever or other malaria symptoms in any health facilities since beginning of July 2014) will be recruited as controls. The assessed exposure is whether the children received SMC, SMC and nutrition products, or neither.

Starting directly after the first administration period, cases and controls will be recruited from health facilities. Information from both cases and controls will be collected through a short (about 30 minutes), pre-tested, malaria-case tracker that covers demographics (age, gender, ward of residence), malaria symptoms and severity, and nutrition indicators, and intervention exposure status (whether the child received SMC or nutrition products). Health care workers will be trained on case eligibility criteria and will collect the data on the child from the parent/guardian. Health care workers will be given a job aid that will assist them in classifying severe versus uncomplicated malaria based on the child's symptoms using the Nigerian National Policy. Data from the tracker will be aggregated by a trained health care

worker on a weekly basis and collected by CHAI staff through routine visits. Small facilitations fees will be given to health facilities for participation.

Assuming intervention coverage in the population of ~60% and a detectable odds-ratio of 0.6 (odds of receiving SMC + LNS among malaria cases compared to SMC alone), we would need to recruit 326 cases (aged 6 to 24 months) with a power of 90% and alpha of 5%. To increase power, controls will be selected at a ratio of 4 controls:1 case, meaning we will select 1,304 controls. Assuming approximately on average 5-6 confirmed cases of malaria, per health facility per month, recruitment will occur in 10 health facilities over a 6 months period August 2014-January2015.

Case-control inclusion criteria are that the child must:

1.) Reside in the intervention catchment area
2.) Be 6-24 months of age at the start of the intervention (July 2014)
3.) Have a parent/guardian that provided informed consent (for parents/guardians who are between the ages of 12-18 they must provide informed assent and if their parent/guardian is nearby, this parent/guardian must also provide informed consent form)
4.) Must be eligible for SMC/LNS delivery (see eligibility criteria on pages 11-14)
5.) Cases must be parasitologically confirmed by either RDT or microscopy
6.) Controls must have had no previous malaria symptoms (no fever in the last 4-weeks) and no care was sought for fever or other malaria symptoms in any health facilities for them since beginning of July 2014)

Focus Group Discussions

In order to identify the barriers and facilitators to SMC and LNS delivery and provide context to the study, 6 FGDs will be completed. FGDs will occur among parents/guardians and CDDs, three FGDs in each intervention area (2 per arm with caregivers and 1 with distributors). Ward officials will be asked to recruit participants. Ward officials will be asked to select a mix of individuals who represent a diverse range of knowledge, attitudes, and practices and would be willing to participate. It is expected that focus group discussions will take between 45-60 minutes and light refreshments will be provided afterwards. Questions will be targeted at strengthening CHAI's understanding of perceptions, challenges, and provide ideas for providing recommendations for co-packaging SMC + nutrition interventions and will occur in Hausa. The FGDs could also provide context to the malnutrition indicators and intra-household nutrition equity and distribution. Every participant must be over the age of 18 and have provided inform consent prior to participation.

Monitoring Data Collection Procedures and Participant Recruitment

The following 5 programmatic data forms will be created and used in this study.

Stock Logbooks

Each CDD will be provided a stock log at training and will be instructed on how to fill in the stock register. As part of their involvement in this study, they will be requested to ensure a daily stock log is completed tracking daily availability of SP-AQ. If they do not feel able to complete the stock logs, they

will not be able to participate in administration. It is expected to take less than 5 minutes per day to fill out the register. Study staff will return after each administration round to collect data. The purpose of the stock logs is to provide on-going data on the number of treatments administered and remaining with CDDs in each treatment area.

Treatment Registers

Each CDD will be provided a treatment register at training and will be instructed on how to fill in the register. Every child that receives a dose of SP-AQ or SP-AQ + LNS will need to be entered into the treatment register before administration. As part of their involvement in this study, they will be requested to record the name, age, sex, and contact details of each child that they administer treatment to. If they do not feel able to complete the treatment register, they will not be able to participate in in administration. It is expected to take less than 1 minute per patient to fill out the register. Study staff will collect the books after administration round 4. The purpose of the treatment registers is to provide on-going data on the number of treatments administered and to whom in each intervention area and assist with the development of the sampling frame for the control portion of the case-control study.

Treatment Cards

Every child 3-59 months in July 2014 reached by a CDD will be assigned an ID and their parent/guardian will be given a treatment card. Each time a child receives a treatment their parent/guardian will be asked to provide their treatment card and the CDD will record the dose and date of administration on the child's card as a record of the child's participation in the study. If the child is ineligible for treatment, the CDD will also record this on treatment card. The parent will be asked to keep the card in a safe place. The purpose of the treatment card is to provide a record of what treatment was provided, to which child, and the date of administration. It is both a record for the parent and will be also be referred to during the household survey.

Referral Forms

During any round of administration or data collection activity, any child found to be severely malnourished will be recorded on the nutrition referral form and immediately be referred to the nearest CNAM for treatment.

Drug-Adverse Reactions Form

For any child that experiences a severe reaction to either SMC or LNS a Drug Adverse Reactions Form (ARF) will be completed and the child will be referred to the nearest public health facility for further observation or treatment. Additional details are included in the *Potential Risks to Participants* section on pages 19-20.

6 Ethical Considerations

This study will be submitted to the Research and Ethics Committee of Kano State Ministry of Health, under the Hospitals Management Board, for approval. Further clearance to conduct the study will be sought from the KSMOH through the KSMCP and from the District Health Officers (DHOs) in the selected

wards. Informed consent will be collected from all participants enrolled in the study prior to their inclusion.

Potential Risks to Participants

While SP+AQ is safe and well tolerated when used in recommended doses and regimens mild side effects may occur of which the most common is vomiting associated with AQ ingestion. Severe side effects including severe skin reactions and blood dyscrasias are rare. In Senegal nearly 800,000 treatment courses of SMC with SP+AQ were given to children; no serious adverse events attributable to SMC drugs were detected despite a high level of surveillance. To mitigate side effects, children will receive SP-AQ with purified water and parents/guardians will be instructed to have fed the child prior to drug administration. Each child will be observed for 15 minutes post-ingestion for signs of dangerous side effects. Drug administrators will be trained and well versed on the signs of danger to watch for; the consent form will also outline this to the child's parent/guardian. At the first sign of a life-threatening adverse reaction the child will be immediately referred to the nearest health center. Emphasis will be given to the voluntary nature of participation in the consent form and during the study. Participants will be informed that they are free to discontinue participation at anytime and that whether or not they participate will not affect their ability to get treatment in health facilities or access other services within the community in the future.

Similarly, with Plumpy'Doz™, while unlikely, there is a small chance of a child having an allergic reaction to the peanut component of the product. The allergy may cause reactions in the form of: skin changes (hives, rashes and infections); body swelling; shortness of breath; and anaphylactic shock. To monitor potential side effects during the first ingestion of Plumpy'Doz™ each child will be observed for 15 minutes post-ingestion for signs of dangerous side effects. Drug administrators will be trained and well versed on the signs of danger to watch for; the consent form will also outline this to the child's parent/guardian. At the first sign of an allergic reaction or a life-threatening adverse reaction the child will be immediately referred to the nearest health center. Emphasis will be given to the voluntary nature of participation in the consent form and during the study. Participants will be informed that they are free to discontinue participation at anytime and that whether or not they participate will not affect their ability to get treatment in health facilities or access other services within the community in the future.

In addition to routine monitoring, as part of routine surveillance, any child who experiences adverse reactions will be recorded on a drug adverse reaction form. For severe drug adverse reactions, the child will also be referred to the nearest public health facility. All local health facilities as well as CDDs will be briefed on the reporting procedure.

Table 5: Reporting Procedure for Severe Adverse Reaction.

Step 1	TREATMENT (Discontinue drug and manage symptoms, e.g. through use of systemic corticosteroids such as hydrocortisone to accelerate recovery in severe cases)

Step 2	Complete the Drug-Adverse Reactions Form (ARF)
Step 3	Immediately contact CHAI study staff using the phone number listed on the (ARF)
Step 4	CHAI study staff will record the event and alert NAFDAC

The greatest risk to participants that participate in the FGDs, interviews, or fill in registers and stock logs are the time required. The participants will be asked where they wish to take the survey, which will help to ensure that they are comfortable and that their privacy is maintained. All data collection activities will be kept as brief as possible and will only be collected from those that have provided informed consent. Participation in any data collection activity will be voluntary.

Potential Benefits to Participants

Children who receive SP-AQ will be at significantly lower risk for malaria transmission and the associated risks of malaria. Households that participate in the SP-AQ administration will be at lower risk for the economic loss associated with malaria sickness within the household. Children who receive LNS will be at significantly lower risk for malnutrition and the associated risks of being immuno-compromised as a result of malnutrition. Households that participate in the SP-AQ +LNS administration will be at lower risk for the economic loss associated with sickness within the household. All CDDs and health facilities recruiting cases for the case control study will receive a small facilitation fee for their contributions to the study.

Informed Consent and Participant Rights

Informed consent will be sought from all study participants before treatment administration or any survey or focus group activity is conducted. For consent, CHAI study staff will provide the consent forms to participants to read and sign with either their signature or thumbprint. The study staff will read aloud to participants the informed consent in the respondent's primary language to ensure understanding of the information within it. The consent statement introduces the broad concept research, outlines the risks and benefits to participating, assures that confidentiality will be maintained, and makes clear that participants have a choice about whether to participate and that they may withdraw at any time. A copy of the consent form they have signed with contact details of the study coordinator and Internal Review Board (IRB) will be provided to them.

Data Management and Confidentiality

All data, which contains identifying information such as names, will be de-identified by assigning individuals IDs. No other identifying information will be included in computer databases. Data from household surveys will be collected electronically by using android tablet devices. The malaria case tracker will be recorded on paper entered into a database using CSPro on a password-protected computer. FGDs will be taped and transcribed. All paper documents with identifying including field notebooks, treatment registers, stock logs, malaria case tracker, and FGDs transcripts will be stored in a

secured cabinet in the CHAI-Nigeria office in Abuja for the duration of the analysis and data containing identifiers will be destroyed upon completion of the analysis.

Only study staff represented in this protocol and the data collection agency will have access to the data containing identifying characteristics. The participants will be given a choice to provide voluntary informed consent before participating in any data collection activity in order to assure that the confidentiality of collected data is maintained. When information is shared with anyone, including participants, non-governmental organizations, governmental institutions and the research community, no names or other identifying information will be included.

7 Dissemination Plan

Monthly updates on the study and available preliminary results will be presented to the KSMCP/SMOH. Upon completion of the study, results will be summarized in a report which will be presented alongside a PowerPoint to the KSMCP and relevant stakeholders. To disseminate the results of the study to the scientific community, aspects of the study will be submitted for publication to peer-reviewed journals, and abstracts submitted to relevant conferences for presentation.

8 Potential for Policy Impact

Findings from this study will allow the KSMCP and the other eight States suitable for SMC + LNS in Nigeria to make a decision regarding whether LNS should be delivered through the SMC campaigns. Additionally, the results will be beneficial to inform the global malaria policy community about how to scale up SMC + LNS in other suitable settings.
