# Supplementary material for: Seasonal malaria chemoprevention packaged with malnutrition prevention in northern Nigeria: A pragmatic trial (SMAMP study) with nested case-control
Source: PLoS One. 2019 Jan 25;14(1):e0210692. doi: 10.1371/journal.pone.0210692 (PMC6347255; doi:10.1371/journal.pone.0210692)
Supplement: S2 File — (DOCX) [file pone.0210692.s007.docx]

| 2. Child Information | | 3. Malaria | | 4. Seasonal Malaria Chemoprevention | | | 6. Anthropometry *(If >10% difference between Measurement 1 & 2, take Measurement 3. Otherwise leave blank)* | | |  |
| --- | --- | --- | --- | --- | --- | --- | --- | --- | --- | --- |
| **2.1** | **Was consent given to participate in the case-control study?**  Yes 1  No 2 | **3.1** | **Was a Rapid Diagnostic Test (RDT) used to test for malaria?**  Yes 1  No 2 | **4.1** | **Did the child receive SPAQ for malaria prevention in the last 30 days?** *Use job aid for reference.*  Yes 1 What was the date? [__\|__] / [__\|__] / [__\|__\|__\|__]  D D M M Y Y Y Y  No 2 If 4.1 is no, enter 99/99/9999 | | **6.1** | | **Mid-upper arm circumference, in centimeters, with a precision of 0.1cm (1 digit beyond period).**  Measurement 1 [_____\|_____].[_____] cm  Measurement 2 [_____\|_____].[_____] cm  Measurement 3 [_____\|_____].[_____] cm  *Measurement not taken = 99.9* |  |
| **2.2** | **Record the child’s full name**  [ ] |  |  | **4.2** | **If child did NOT receive SPAQ in last 30 days, then circle all that apply:**  Did not know about SMC A  Not accessible/available B  Ineligible because sick with fever or malaria C  A distributor did not delivery to my house D  Fear it is poison or will make child sick E  No malaria risk F  Head of household forbids it G  Do not give pills to child unless sick H  Child developed allergy to SMC I  Do not like SMC J  Was not at home/available during distribution K  Child did not like bitter taste L  Child was previously sick from SMC M  Other (specify) X  Refused Y  Don’t Know Z  If 4.1 is Yes then leave blank | |  |  |  |  |
|  |  | **3.2** | **What was the result of the RDT test?**  Positive 1  Negative 2 |  |  |  |  |  |  |  |
| **2.3** | **Record the child’s ID Number**  [____\|____\|____]____] |  |  |  |  |  |  |  |  |  |
|  |  | **3.3** | **What this malaria episode uncomplicated or severe malaria?** *Use job aid for reference*  Uncomplicated 1  Severe 2  Not Applicable (RDT Negative) 96 |  |  |  |  |  |  |  |
| **2.4** | **What is the child’s gender?**  Male 1  Female 2 |  |  |  |  |  | **6.2** | | **Body weight measurement value with a precision of 0.05kg (2 digits beyond period)**  Measurement 1 [___\|___].[___\|___] kg  Measurement 2 [___\|___].[___\|___] kg  Measurement 3 [___\|___].[___\|___] kg  Measurement not taken = 99.99 |  |
| **2.5** | **What is the child’s age, in years and months?**  [____] Years, [____\|____] Months | **3.4** | **What was the date the malaria symptoms began?**  [__\|__] / [__\|__] / [__\|__\|__\|__]  D D M M Y Y Y Y  Not applicable (RDT Negative) 99/99/999 |  |  |  |  |  |  |  |
| **2.6** | **What is the entry date?**  [__\|__] / [__\|__] / [__\|__\|__\|__]  D D M M Y Y Y Y | **3.5** | **What other illnesses did the child have in the past 2 weeks, if any?** *Circle all that apply*  None A  Cold/Flu B  Diarrhea/Dehydration C  Pneumonia D  Respiratory Disease E  Measles F  Chicken Pox G  Meningitis (brain infection) H  Tuberculosis I  Cholera J  Typhoid Fever K  Yellow Fever L  Cardiovascular Disease M  Parasite (schistosomiasis/helminth) N  Polio O  HIV/AIDS P  Other (Specify) X  Refused Y  Don’t Know Z | **4.3** | **How many days of the SP-AQ treatment regimen did the child take during the latest round of distribution?**  One day 1  Two days 2  Three days 3  None 4  Not Applicable (4.1 is NO) 96  Refused 98  Don’t Know 99 | |  | | **Record height measurement value with a precision of 0.1cm (1 digit beyond period)**  Measurement 1 [___\|___\|___].[___] cm  Measurement 2 [___\|___\|___].[___] cm  Measurement 3 [___\|___\|___].[___] cm  Measurement not taken = 999.9 |  |
|  |  |  |  |  |  |  | **6.3** | |  |  |
| **2.7** | **Record child’s ward and village residence**  Ward name [ ]  Ward code [____\|____]  Village name[ ] |  |  |  |  |  |  |  |  |  |
| **2.8** | **How can you tell if someone has malaria?** *Do not prompt. Probe: Any others? Circle all that apply.*  Fever A  Sweating B  Headache C  Chills/Shivering D  Poor appetite E  Vomiting F  Cough/Difficulty breathing G  Diarrhea H  Joint pain I  Convulsion J  Coma K  Other (specify) X  Refused Y  Don’t Know Z |  |  | 5. Lipid-based Nutritional Supplement *(for 5.7 and 5.8 if Not Applicable, then enter code 96)* | | | | | |  |
|  |  |  |  | **5.1** | **Since August 2014, did the** Yes 1  **child ever receive Plumpy’doz?** No 2 | **5.5** | | **How many times per week did**  **the child consume Plumpy’doz** [ _ _\|_ _ ] times  **over the past month?** Not applicable 96 | | |
|  |  |  |  | **5.2** | **From when did the child If 5.1 is NO, then**  **start taking Plumpy’doz? enter**  [___\|___] / [___\|___] / [___\|___\|___\|___] 99/99/999  D D M M Y Y Y Y | **5.6** | | **Yesterday, during the day** Yes 1 No 2  **or night, did the child** Not Applicable 96  **consume any Plumpy’doz?** Refused 98  Don’t Know 99 | | |
|  |  | **3.6** | **Did the child sleep under a** Yes 1  **bednet in the past 2 weeks?** No 2 | **5.3** | **Did the child ever stop taking Plumpy’doz since this date?**  Yes 1 No 2  Not Applicable 96 | **5.7** | | **Yesterday, how many times** Times [___\|___]  **did the child eat Plumpy’doz**  Refused 98  **during the day or at night?**  Don’t Know 99 | | |
|  |  | **3.7** | **In the past 12 months, was your**  Yes 1  **house sprayed with chemicals to**  No 2  **protect from malaria?** | **5.4** | **If the child stopped taking** If not applicable  **Plumpy’doz, when did they stop?** then enter [__\|___] / [___\|___] / [___\|___\|___\|___] (DD/MM/YYYY) 99/99/9999 | **5.8** | | **In total how much of**  Teaspoons [___\|___]  **Plumpy’doz did the child** Refused 98  **consume yesterday?** Don’t Know 99 | | |
